# Understanding network concepts in modules

**DOI:** 10.1186/1752-0509-1-24

**Published:** 2007-06-04

**Authors:** Jun Dong, Steve Horvath

**Affiliations:** 1 Department of Human Genetics and Department of Biostatistics, University of California, Los Angeles, CA 90095, USA

## Abstract

**Background:**

Network concepts are increasingly used in biology and genetics. For example, the clustering coefficient has been used to understand network architecture; the connectivity (also known as degree) has been used to screen for cancer targets; and the topological overlap matrix has been used to define modules and to annotate genes. Dozens of potentially useful network concepts are known from graph theory.

**Results:**

Here we study network concepts in special types of networks, which we refer to as approximately factorizable networks. In these networks, the pairwise connection strength (adjacency) between 2 network nodes can be factored into node specific contributions, named node 'conformity'. The node conformity turns out to be highly related to the connectivity. To provide a formalism for relating network concepts to each other, we define three types of network concepts: fundamental-, conformity-based-, and approximate conformity-based concepts. Fundamental concepts include the standard definitions of connectivity, density, centralization, heterogeneity, clustering coefficient, and topological overlap. The approximate conformity-based analogs of fundamental network concepts have several theoretical advantages. First, they allow one to derive simple relationships between seemingly disparate networks concepts. For example, we derive simple relationships between the clustering coefficient, the heterogeneity, the density, the centralization, and the topological overlap. The second advantage of approximate conformity-based network concepts is that they allow one to show that fundamental network concepts can be approximated by simple functions of the connectivity in module networks.

**Conclusion:**

Using protein-protein interaction, gene co-expression, and simulated data, we show that a) many networks comprised of module nodes are approximately factorizable and b) in these types of networks, simple relationships exist between seemingly disparate network concepts. Our results are implemented in freely available R software code, which can be downloaded from the following webpage: http://www.genetics.ucla.edu/labs/horvath/ModuleConformity/ModuleNetworks

## Background

Network terminology is used to study important questions in systems biology. For example, networks are used to study functional enrichment [1], to analyze the structure of cellular networks [2], to model biological signalling or regulatory networks [1,3], to reconstruct metabolic networks [4], and to study the dynamic behavior of gene regulatory networks [5].

Here we study the meaning of network concepts in relatively simple networks, e.g. gene co-expression networks and protein-protein interaction (PPI) networks. Specifically, we consider undirected networks that can be represented by a symmetric adjacency matrix *A *= [*a*_*ij*_], where the pairwise adjacency (connection strength) *a*_*ij *_takes values in the unit interval, i.e., 0 ≤ *a*_*ij *_≤ 1. For an *unweighted *network, the adjacency *a*_*ij *_= 1 if nodes *i *and *j *are connected and 0 otherwise. For a *weighted *network, 0 ≤ *a*_*ij *_≤ 1. For notational convenience, we set the diagonal elements to 1.

### Fundamental network concepts

Other authors refer to network concepts as network statistics or network indices. Network concepts include connectivity, mean connectivity, density, variance of the connectivity (related to the heterogeneity) etc. Network concepts can be used as descriptive statistics for networks. While some network concepts (e.g. connectivity) have found important uses in biology and genetics, other network concepts (e.g. network centralization) appear less interesting to biologists. Before attempting to understand why some concepts are more interesting than others, it is important to understand how network concepts relate to each other in biologically interesting networks. As a step toward this goal, we explore the meaning of network concepts in module networks, which are defined below.

In the following, we review fundamental network concepts. Further details on the definitions and notations can be found in the Methods section.

The **node connectivity **is given by

(1) $$Connectivity_{i}=k_{i}=\sum_{j\neq i}a_{ij}.$$

In unweighted networks, the connectivity *k*_*i *_of node *i *equals the number of directly linked neighbors. In weighted networks, the connectivity equals the sum of connection weights with all other nodes. Highly connected 'hub' genes are thought to play an important role in organizing the behavior of biological networks [6-9]. Connectivity has been found to be an important complementary gene screening variable for finding biologically significant genes in cancer [10,11] and primate brain development [12].

The **line density **[13] is defined as the mean off-diagonal adjacency and is closely related to the mean connectivity.

(2) $$Density=\frac{\sum_{i}\sum_{j\neq i}a_{ij}}{n(n-1)}=\frac{S_{1}(k)}{n(n-1)}=\frac{mean(k)}{n-1},$$

where the function *S*_*p*_(·) is defined for a vector ***v ***as *S*_*p*_(***v***) = ∑_*i*_$v_{i}^{p}$ = (***v***^*p*^)^*τ *^***1***.

The normalized connectivity **centralization **(also known as degree centralization) is a simple and widely used index of the connectivity distribution. By definition [14], the normalized connectivity centralization is given by

(3) $$Centralization=\frac{n}{n-2}\left(\frac{\max(k)}{n-1}-Density\right)\approx \frac{\max(k)}{n}-Density.$$

A frequent question of social network analysis concerns the causes and consequences of centralization in network structure, i.e. the extent to which certain nodes are far more central than others within the network in question. The centralization index has been used to describe structural differences of metabolic networks [15].

Many measures of network heterogeneity are based on the variance of the connectivity, and authors differ on how to scale the variance [13]. Our definition of the network **heterogeneity **equals the coefficient of variation of the connectivity distribution, i.e.

(4) $$Heterogeneity=\frac{\sqrt{variance(k)}}{mean(k)}=\sqrt{\frac{nS_{2}(k)}{S_{1}{\left(k\right)}^{2}}-1}.$$

This heterogeneity measure is scale invariant with respect to multiplying the connectivity by a scalar. Biological networks tend to be very heterogeneous: while some 'hub' nodes are highly connected, the majority of nodes tend to have very few connections. Describing the heterogeneity (inhomogeneity) of the connectivity (degree) distribution has been the focus of considerable research in recent years [6,16-18].

The **clustering coefficient **of node *i *is a density measure of local connections, or 'cliquishness' [19,20]. Specifically,

(5) $$ClusterCoef_{i}=\frac{n_{i}}{\pi_{i}}=\frac{\sum_{l\neq i}\sum_{m\neq i,l}a_{il}a_{lm}a_{mi}}{\left\{{\left(\sum_{l\neq i}a_{il}\right)}^{2}-\sum_{l\neq i}a_{il}^{2}\right\}}.$$

In unweighted networks, *n*_*i *_equals twice the number of direct connections among the nodes connected to node *i*, and *π*_*i *_equals twice the maximum possible number of direct connections among the nodes connected to node *i*. Consequently, *ClusterCoef*_*i *_equals 1 if and only if all neighbors of *i *are also connected to each other. For general weighted networks with 0 ≤ *a*_*ij *_≤ 1, one can prove 0 ≤ *ClusterCoef*_*i *_≤ 1 [21]. The relationship between the clustering coefficient and modular structure has been investigated by several authors [20,22-24].

The **topological overlap **between nodes *i *and *j *reflects their relative interconnectedness [20,25]. It is defined by

(6) $$TopOverlap_{ij}=\frac{l_{ij}+a_{ij}}{\min\left\{k_{i},k_{j}\right\}+1-a_{ij}},$$

where *l*_*ij *_= ∑_*u*≠*i*,*j*_*a*_*iu*_*a*_*uj*_. In an unweighted network, *l*_*ij *_equals the number of nodes to which both *i *and *j *are connected. In this case, *TopOverlap*_*ij *_= 1 if the node with fewer connections satisfies two conditions: (a) all of its neighbors are also neighbors of the other node, and (b) it is connected to the other node. In contrast, *TopOverlap*_*ij *_= 0 if *i *and *j *are un-connected and the two nodes do not share any neighbors. By convention, *TopOverlap*_*ii *_= 1. One can prove that 0 ≤ *a*_*ij *_≤ 1 implies 0 ≤ *TopOverlap*_*ij *_≤ 1 [21].

#### The Topological Overlap Matrix Can Be Considered as Adjacency Matrix

Since the matrix *TopOverlap *= [*TopOverlap*_*ij*_] is symmetric and its entries lie in [0, 1], it satisfies our assumptions on an adjacency matrix. Roughly speaking, the topological overlap matrix can be considered as a 'smoothed out' version of the adjacency matrix. The elements of *TopOverlap *provide an alternative measure of connection strength based on shared neighbors. There is evidence that replacing *A *by *TopOverlap *may counter the adverse effects of spurious or missing adjacencies [25,26]. Since the adjacency matrices of the PPI networks in our applications were very sparse, we replaced them by the corresponding topological overlap matrices. In contrast, we used the original adjacency matrix when analyzing gene co-expression networks since high specificity is desirable for measuring interconnectedness in co-expression networks.

### The topological overlap matrix can be used for module definition

Our main interest lies in (sub-)networks comprised of nodes that form a module inside a larger network. Since a particular module network may encode a pathway or a protein complex, these special types of networks have great practical importance. Similar to the term 'cluster', no consensus on the meaning of the term 'module' seems to exist in the literature. In our applications, we use a clustering procedure to identify modules (clusters) of nodes with high topological overlap. We follow the suggestion of [20] to turn the topological overlap matrix *TopOverlap *into a *dis*similarity measure by subtracting it from 1, i.e. *dissTopOverlap*_*ij *_= 1 - *TopOverlap*_*ij*_.

**Figure 1:**
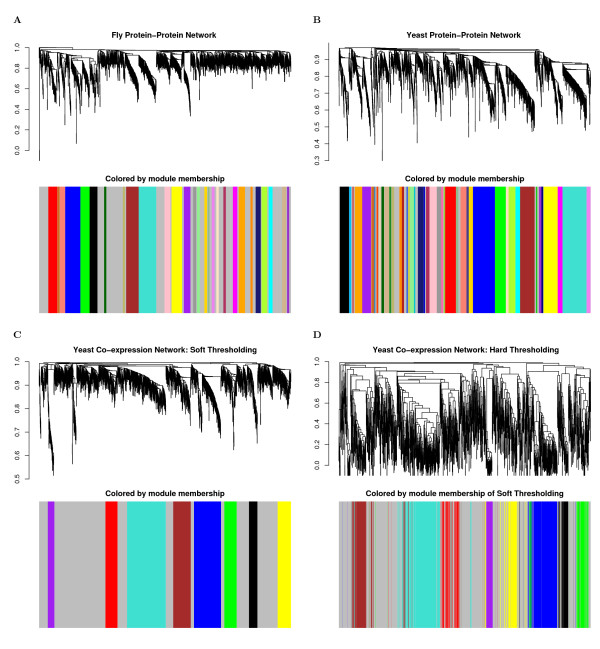
**Hierarchical clustering dendrogram and module definition**. A) Drosophila PPI network. The dendrogram results from average linkage hierarchical clustering. The color-band below the dendrogram denotes the modules, which are defined as branches in the dendrogram. Of the 1371 proteins, 862 were clustered into 28 proper modules, and the remaining proteins are colored in grey; B) yeast PPI network; C) weighted gene co-expression network (yeast); D) unweighted gene co-expression network (yeast). To facilitate a comparison between the weighted and the unweighted gene co-expression networks, we used the module assignment of C) in D). Note that the colors of C) tend to stay together in D), which illustrates high module preservation.

We use *dissTopOverlap*_*ij *_as input of average linkage hierarchical clustering to arrive at a dendrogram (clustering tree) [27]. Modules are defined as the branches of the dendrogram. For example, in Figure 1 we show the dendrograms of our network applications. Genes or proteins of proper modules are assigned a color (e.g. turquoise, blue etc). Genes outside any proper module are colored grey. Our module definition depends on how the branches are cut off the dendrogram. Several methods and criteria for identifying branches in a dendrogram have been proposed, see e.g. [20,21,28]. In practice, it is advisable to study how robust the results are with respect to alternative module detection methods. In our online R software tutorial, we show that our findings are highly robust with respect to alternative module definitions. In addition, we use a functional enrichment analysis of the resulting modules to provide indirect evidence that the modules are biologically meaningful. Our module detection approach has led to biologically meaningful modules in several applications [9,10,12,20,28-30] but we make no claim that it is optimal. Our theoretical results will apply to all module detection methods that result in approximately factorizable networks.

## Results

### Conformity and factorizable networks

We define an adjacency matrix *A *to be exactly factorizable if, and only if, there exists a vector ***CF*** with non-negative elements such that

(7) *a*_*ij *_= *CF*_*i*_*CF*_*j *_   for all   *i *≠ *j*

If the non-negative solution of equation (7) is unique, it is referred to as conformity vector ***CF*** and *CF*_*i *_is the conformity of node *i*. One can easily show that the vector ***CF ***is not unique if the network contains only *n *= 2 nodes. However, for *n *> 2 it is unique for a weighted network, see our derivations surrounding equation (20).

We also define the concept of conformity for a general, non-factorizable network. The idea is to find an exactly factorizable adjacency matrix *A*_*CF *_= ***CF CF***^*τ *^- *diag*(***CF***^2^) + *I *****that best approximates *A*. Note that the diagonal elements of *A*_*CF *_and *A *equal 1.

In the appendix, we define the conformity as a maximizer of the factorizability function $F_{A}(v)=1-\frac{\sum_{i}\sum_{j\neq i}{\left(a_{ij}-v_{i}v_{j}\right)}^{2}}{\sum_{i}\sum_{j\neq i}{\left(a_{ij}\right)}^{2}}$. Alternative methods of decomposing an adjacency matrix are briefly discussed below.

In equation (43), we define a measure of network factorizability as follows

$$F(A)=1-\frac{{\left\|\left(A-I)-(A_{CF}-I\right)\right\|}_{F}^{2}}{{\left\|A-I\right\|}_{F}^{2}}.$$

The factorizability *F*(*A*) is normalized to take on values in the unit interval [0, 1]. The higher *F*(*A*), the better *A*_*CF *_- *I *approximates *A *- *I*.

### Modules can be approximately factorizable

Approximate factorizability is a very strong structural assumption on an adjacency matrix. It certainly does not hold for general networks. However, we provide empirical evidence that many clusters (modules) of genes or proteins in real networks are approximately factorizable. Table 1 reports the mean values of *F*(*A*) for the applications considered in this paper. For example in the Drosophila PPI network, the mean factorizability *F*(*A*) is 0.82 across 'proper' modules defined as clusters in the network. In contrast, the factorizability of the subnetwork comprised of non-module nodes is only 0.17. In the yeast PPI network, the mean factorizability of proper modules is 0.85 while it equals only 0.20 for the grey module. In the weighted yeast gene co-expression network, the mean factorizability of proper modules equals 0.73 while it is only 0.18 for the improper module. Similarly in the unweighted yeast gene co-expression network, the mean factorizability of proper modules equals 0.62 while it is only 0.11 for the improper module. A more detailed table presenting network concepts in each module is also provided [see Additional file 1].

**Table 1:** Summary of fundamental network concepts in real network applications.

	Fly Protein		Yeast Protein		Yeast (Weighted)		Yeast (Unweighted)	
				
Concept	Proper	Grey	Proper	Grey	Proper	Grey	Proper	Grey
Factorizability	.82 (.086)	.170	.85 (.100)	.200	.73 (.084)	.180	.62 (.130)	.110
Density	.21 (.074)	.017	.28 (.120)	.026	.08 (.056)	.005	.40 (.150)	.024
Centralization	.18 (.091)	.052	.20 (.055)	.036	.10 (.026)	.021	.41 (.110)	.140
Heterogeneity	.35 (.130)	.460	.36 (.140)	.430	.56 (.066)	.580	.51 (.097)	.830
Mean Cluster Coef.	.28 (.110)	.050	.36 (.120)	.093	.13 (.072)	.032	.72 (.087)	.370
Mean Conformity	.45 (.076)	.130	.51 (.120)	.150	.26 (.084)	.062	.63 (.100)	.120

Each network contained several proper modules. Non-module genes were grouped into a single (improper) grey module. For each concept, we report the mean and standard error across the proper modules. A more detailed table presenting network concepts in each module is also provided [see Additional file 1].

Our empirical results support the following

**Observation 1 ***For many modules defined with a clustering procedure, the subnetwork comprised of the module nodes is approximately factorizable.*

This observation motivates us to study network concepts in approximately factorizable networks.

### Conformity-based network concepts

We refer to the standard network concepts known from the literature as *fundamental *network concepts. In general, fundamental network concepts are functions of the off-diagonal elements of the adjacency matrix *A*. More precisely, we use *network concept functions *to define different types of network concepts depending on the input matrix (see Table 2 and equation (21)). For example, when inputting an adjacency matrix with its diagonal elements replaced by 0, one arrives at fundamental network concepts (see Definition 5 in the Methods section). When inputting the conformity-based (CF-based) adjacency matrix *A*_*CF *_with its diagonal elements replaced by 0, one arrives at CF-based network concepts (see Definition 6 in the Methods section). The conformity vector can be used to define the approximate CF-based matrix

**Table 2:** Brief overview of different types of network concepts.

Input Matrix	Type of Concept	Example: Connectivity
*A *- *I*	fundamental	*Connectivity*_*i*_(*A *- *I*) = ∑_*j*≠*i*_*a*_*ij*_
*A*_*CF *_- *I *= ***CF CF***^*τ *^- diag(***CF***^2^)	CF-based	*Connectivity*_*i*_(*A*_*CF *_- *I*) = *CF*_*i*_∑_*j*≠*i*_*CF*_*j*_
*A*_*CF*,*app *_= ***CF CF***^*τ*^	approximate CF-based	*Connectivity*_*i*_(*A*_*CF*__,__*app*_) = *CF*_*i*_∑_*j*_*CF*_*j*_

A network concept arises by evaluating a *network concept function *on a special type of input matrix. We assume that the diagonal elements of the matrix *A *- *I *are 0.

*A*_*CF*,*app *_= ***CF CF***^*τ *^= [*CF*_*i*_*CF*_*j*_].

Note that the *i*-th diagonal element of *A*_*CF*,*app *_equals $CF_{i}^{2}$. When *A*_*CF*,*app *_is used as input of a network concept function, one arrives at an approximate CF-based concept (see Definition 7 in the Methods section).

We will demonstrate that approximate CF-based concepts satisfy simple relationships. Below, we show that these simple relationships carry over to fundamental network concepts in approximately factorizable networks.

In Definition 7, we provide a formula for calculating approximate CF-based analogs of the fundamental network concepts. Specifically, we find

(8) $$\begin{array}{lll} k_{CF,app,i} & = & CF_{i}S_{1}(CF),\\ Density_{CF,app} & = & \frac{S_{1}{\left(CF\right)}^{2}}{n(n-1)}\approx {\left(\frac{S_{1}(CF)}{n}\right)}^{2},\\ Centralization_{CF,app} & = & \frac{nS_{1}(CF)}{\left(n-1)(n-2\right)}\left(\max(CF)-\frac{S_{1}(CF)}{n}\right)\\ & \approx & \frac{S_{1}(CF)}{n}\left(\max(CF)-\frac{S_{1}(CF)}{n}\right),\\ Heterogeneity_{CF,app} & = & \sqrt{\frac{nS_{2}(CF)}{{\left(S_{1}(CF)\right)}^{2}}-1},\\ ClusterCoef_{CF,app,i} & = & {\left(\frac{S_{2}(CF)}{S_{1}(CF)}\right)}^{2},\\ TopOverlap_{CF,app,ij} & = & \frac{CF_{i}CF_{j}(S_{2}(CF)+1)}{\min(CF_{i},CF_{j})S_{1}(CF)+1-CF_{i}CF_{j}}, \end{array}$$

where *S*_*p*_(***CF***) = ∑_*i*_(*CF*_*i*_)^*p*^. Note that the approximate CF-based clustering coefficient does not depend on the *i*-index. This is why we sometimes omit this index and simply write *ClusterCoef*_*CF*,*app*_.

### Approximate CF-based network concepts satisfy simple relationships

Here we demonstrate a major advantage of approximate CF-based network concepts: they exhibit simple relationships. Using the fact that *S*_1_(***k***_*CF*,*app*_) = *S*_1_(***CF***)^2^, and the approximation *n*/(*n *- 1) ≈ 1, equations (8) imply the following relationship

$$Heterogeneity_{CF,app}\approx \sqrt{\sqrt{\frac{ClusterCoef_{CF,app}}{Density_{CF,app}}}-1},$$

or equivalently,

(9) $$ClusterCoef_{CF,app,i}\approx {\left(1+Heterogeneity_{CF,app}^{2}\right)}^{2}\times Density_{CF,app}.$$

Further, it is straightforward to derive a simple relationship between approximate CF-based topological overlap, connectivity and heterogeneity under the following mild assumptions: $\frac{1}{S_{2}(CF)}\approx 0$ and $\frac{1-CF_{i}CF_{j}}{\min(CF_{i}\text{,}CF_{j})S_{1}(CF)}\approx 0$. Specifically, we find

(10) $$\begin{array}{lll} TopOverlap_{CF,app,ij} & \approx & \max(CF_{i}\text{,}CF_{j})\frac{S_{2}(CF)}{S_{1}(CF)}=\frac{\max(CF_{i}S_{1}(CF),CF_{j}S_{1}(CF))}{n}\frac{nS_{2}(CF)}{S_{1}{\left(CF\right)}^{2}}\\ & \approx & \frac{\max(k_{CF,app,i},k_{CF,app,j})}{n}(1+Heterogeneity_{CF,app}^{2}). \end{array}$$

In the following subsection, we outline the conditions when equations (9) and (10) hold approximately for fundamental network concepts in approximately factorizable module networks.

### Relating fundamental- to approximate CF-based concepts

In the Methods section, we provide a heuristic argument for the following

**Observation 2 ***In approximately factorizable networks, fundamental network concepts are approximately equal to their approximate CF-based analogs,*

*FundamentalNetworkConcept ≈ NetworkConcept*_*CF*,*app*_.

**Figure 2:**
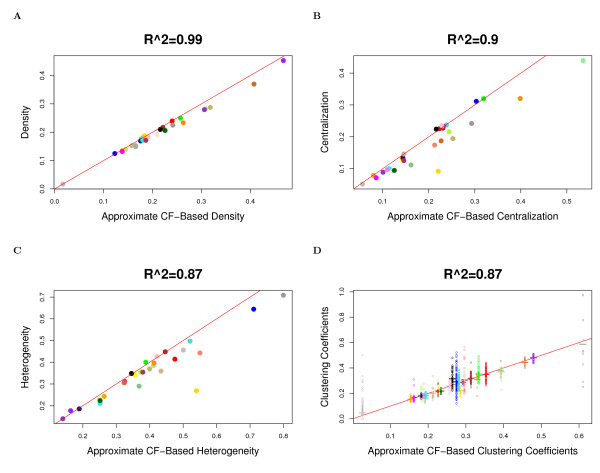
**Drosophila PPI module networks: the relationship between fundamental network concepts *NetworkConcept*(*A *- *I*) (y-axis) and their approximate CF-based analogs *NetworkConcept*_*CF*,*app *_(x-axis)**. This figure demonstrates Observation 2. A) Density versus *Density*_*CF*,*app*_; B) Centralization versus *Centralization*_*CF*,*app*_; C) Heterogeneity versus *Heterogeneity*_*CF*,*app*_; D) Intramodular clustering coefficients *ClusterCoef*_*i *_versus *ClusterCoef*_*CF*,*app*_. In Figures A), B) and C), each dot corresponds to a module since these network concepts summarize an entire network module. In Figure D), each dot corresponds to a node since these network concepts are node specific. A reference line with intercept 0 and slope 1 has been added to each plot.

**Figure 3:**
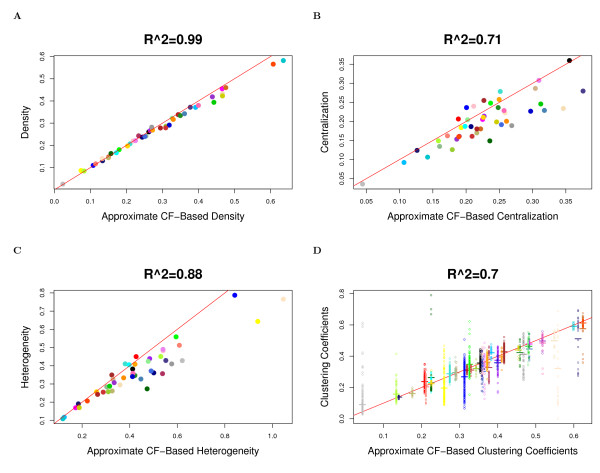
**Yeast PPI module networks: the relationship between fundamental network concepts *NetworkConcept*(*A *- *I*) (y-axis) and their approximate CF-based analogs *NetworkConcept*_*CF*,*app *_(x-axis)**. This figure demonstrates Observation 2. A) Density versus *Density*_*CF*,*app*_; B) Centralization versus *Centralization*_*CF*,*app*_; C) Heterogeneity versus *Heterogeneity*_*CF*,*app*_; D) Intramodular clustering coefficients *ClusterCoef*_*i *_versus *ClusterCoefC*_*F*,*app*_. In Figures A), B) and C), each dot corresponds to a module since these network concepts summarize an entire network module. In Figure D), each dot corresponds to a node since these network concepts are node specific. A reference line with intercept 0 and slope 1 has been added to each plot.

**Figure 4:**
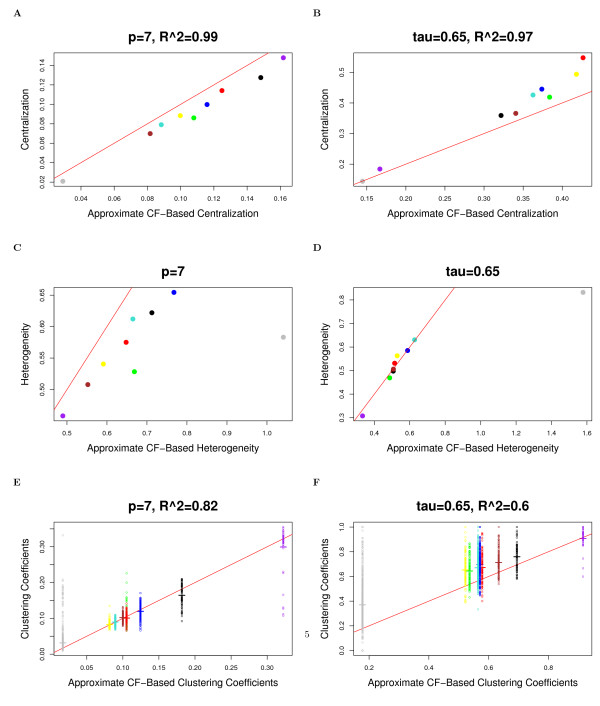
**Yeast gene co-expression module networks: the relationship between fundamental network concepts *NetworkConcept*(*A *- *I*) (y-axis) and their approximate CF-based analogs *NetworkConcept*_*CF*,*app *_(x-axis)**. This figure demonstrates Observation 2. A reference line with intercept 0 and slope 1 has been added to each plot. The figures on the left (right) hand side depict network concepts from the weighted (unweighted) network. A) and B) Centralization versus *Centralization*_*CF*,*app*_; C) and D) Heterogeneity versus *Heterogeneity*_*CF*,*app*_; E) and F) Intramodular clustering coefficients *ClusterCoef*_*i *_versus *ClusterCoef*_*CF*,*app*_. The analogous plots for Density are not presented since the fundamental network concepts and their approximate CF-based analogs are almost identical and the dots fall near the reference line with *R*^2 ^= 1 for both weighted and unweighted networks, and thus are omitted due to limited space. In Figures A), B), C) and D), each dot corresponds to a module since these network concepts summarize an entire network module. In Figure E) and F), each dot corresponds to a node since these network concepts are node specific.

The observation implies that in approximately factorizable networks, *Connectivity ≈ Connectivity*_*CF*,*app *_and *Density *≈ *Density*_*CF*,*app*_, etc. Observation 2 is illustrated for network density, centralization, heterogeneity, and clustering coefficients in Figure 2 (Drosophila PPI network), Figure 3 (yeast PPI network), and Figure 4 (weighted and unweighted yeast gene co-expression networks; density is not included due to limited space). A consequence of this observation is that the simple relationships satisfied by approximate CF-based network concepts also apply to their corresponding fundamental network concepts in approximately factorizable networks. In particular, equations (9) and (10) imply the following

**Observation 3 ***In approximately factorizable networks, the following relationships hold among fundamental network concepts*

(11) *mean*(***ClusterCoef***) ≈ (1 + *Heterogeneity*^2^)^2 ^× *Density*,

and

(12) $$TopOverlap_{ij}\approx \frac{\max(k_{i},k_{j})}{n}(1+Heterogeneity^{2}).$$

**Figure 5:**
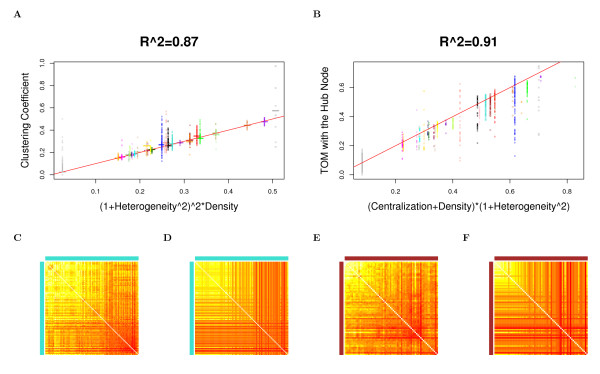
**Drosophila PPI module networks: the relationship between fundamental network concepts**. This figure demonstrates Observation 3 and equation (14). In Figures A) and B), each point is a protein colored by its module assignment, and the red line has intercept 0 and slope 1. Figure A) illustrates the relationship between the mean clustering coefficient (short horizonal line) and (1 + *Heterogeneity*^2^)^2 ^* *Density *(equation (11)). Figure B) illustrates the relationship between the topological overlap with the hub node and (*Density *+ *Centralization*) * (1 + *Heterogeneity*^2^) (equation (14)). Figure C) is a color-coded depiction of the topological overlap matrix *TopOverlap*_*ij *_in the turquoise module network. Figure D) represents the corresponding approximation *max*(*k*_*i*_,*k*_*j*_)(1 + *Heterogeneity*^2^)/*n *(equation (12)). Figures E) and F) are their analogs for the brown module. The turquoise and the brown module represent the largest and third largest module. Analogous plots for the other modules can be found in our online supplement.

**Figure 6:**
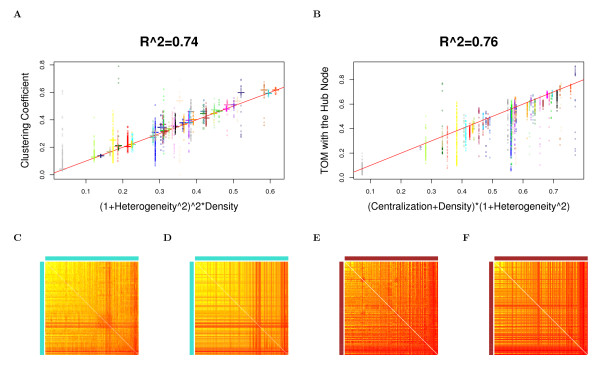
**Yeast PPI module networks: the relationship between fundamental network concepts**. This figure demonstrates Observation 3 and equation (14). In Figures A) and B), each point is a protein colored by its module assignment and the red line has intercept 0 and slope 1. Figure A) illustrates the relationship between the mean clustering coefficient (short horizonal line) and (1 + *Heterogeneity*^2^)^2 ^* *Density *(equation (11)). Figure B) illustrates the relationship between the topological overlap with the hub node and (*Density *+ *Centralization*) * (1 + *Heterogeneity*^2^) (equation (14)). Figure C) is a color-coded depiction of the topological overlap matrix *TopOverlap*_*ij *_in the turquoise module network. Figure D) represents the corresponding approximation *max*(*k*_*i*_,*k*_*j*_)(1 + *Heterogeneity*^2^)/*n *(equation (12)). Figures E) and F) are their analogs for the brown module. The turquoise and the brown module represent the largest and third largest module. Analogous plots for the other modules can be found in our online supplement.

**Figure 7:**
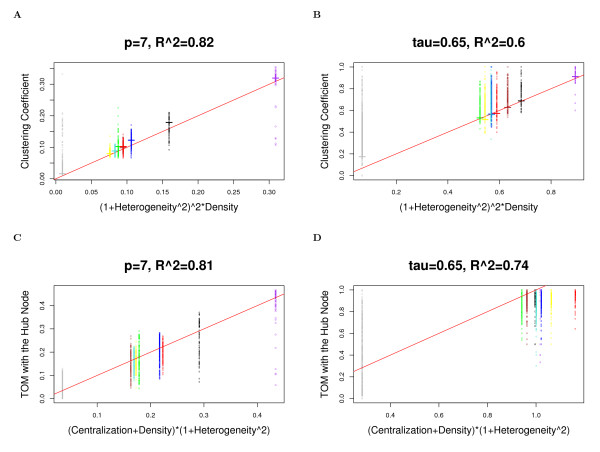
**Yeast gene co-expression module networks: the relationship between fundamental network concepts**. This figure demonstrates Observation 3 and equation (14). The figures on the left (right) hand side depict network concepts from the weighted (unweighted) network. Each point is a gene colored by its module assignment. The red line has intercept 0 and slope 1. Figures A) and B) illustrate the relationship between the mean clustering coefficient (short horizonal line) and (1 + *Heterogeneity*^2^)^2 ^* *Density *(equation (11)). Figure C) and D) illustrates the relationship between the topological overlap with the hub node and (*Density *+ *Centralization*) * (1 + *Heterogeneity*^2^) (equation (14)).

Observation 3 is important since it highlights the fact that seemingly disparate network concepts satisfy simple and intuitive relationships in approximately factorizable networks. Equations (11) and (12) are illustrated in Figure 5 (Drosophila PPI network), Figure 6 (yeast PPI network), and Figure 7 (weighted and unweighted yeast gene co-expression networks; TOM plots are not included due to limited space). Equation (12) has several important consequences. To begin with, it illustrates that the topological overlap between the most highly connected node and all other nodes is approximately constant. Specifically, if we denote the index of the most highly connected node by [1] and its connectivity by *k*_[1] _= *max*(***k***), then

(13) $$TopOverlap_{[1]j}\approx \frac{k_{\left[1\right]}}{n}(1+Heterogeneity^{2}).$$

As an aside, we briefly mention that *TopOverlap*_[1]*j *_has a simple interpretation in terms of the hierarchical clustering dendrogram that results from using *dissTopOverlap*_*ij *_= 1 -*TopOverlap*_*ij *_as input. In this case, *TopOverlap*_[1]*j *_is related to the longest branch length in the dendrogram.

In the following, we relate *TopOverlap*_[1]*j *_to the fundamental network concept *Centralization*. According to equation (3), $\frac{\max(k)}{n}$ ≈ *Centralization *+ *Density*. Substituting this expression in equation (13) implies

(14) *TopOverlap*_[1]*j *_≈ (*Centralization *+ *Density*)(1 + *Heterogeneity*^2^).

Equation (14) is illustrated in Figure 5 (Drosophila PPI network), Figure 6 (yeast PPI network), and Figure 7 (weighted and unweighted yeast gene co-expression networks).

### In factorizable networks, fundamental network concepts are simple functions of the connectivity

Here we demonstrate another advantage of approximate CF-based network concepts. They allow one to relate fundamental network concepts to simple functions of the connectivity. Toward this end, note the following simple relationship between the conformity ***CF ***and the approximate CF-based connectivity ***k***_*CF*,*app*_:

(15) $$CF_{i}=\frac{k_{CF,app,i}}{\sqrt{S_{1}(k_{CF,app})}}.$$

Since in approximately factorizable networks *k*_*CF*,*i *_≈ *k*_*i*_, we find that the conformity ***CF ***is approximately given by the scaled connectivity, i.e.

(16) $$CF\approx \frac{k}{\sqrt{S_{1}(k)}}.$$

This equation shows that conformity can be interpreted as a scaled connectivity in approximately factorizable networks. Since approximate CF-based network concepts are simple functions of the conformity, substituting $\frac{k}{\sqrt{S_{1}(k)}}$ for ***CF ***implies that approximate CF-based concepts can be approximated by simple functions of the connectivity. For example, we find the following simple expressions for the cluster coefficient and the topological overlap.

Observation 4

$$\begin{array}{lll} ClusterCoef_{i} & \approx & \frac{{\left(S_{2}(k)\right)}^{2}}{{\left(S_{1}(k)\right)}^{3}},\\ TopOverlap_{ij} & \approx & \frac{k_{i}k_{j}(S_{2}(k)+S_{1}(k))}{\min(k_{i},k_{j})S_{1}(k)+S_{1}(k)-k_{i}k_{j}}\approx \frac{max(k_{i},k_{j})}{n}\frac{S_{2}(k)}{S_{1}(k)}, \end{array}$$

*where the last approximation assumes *$\frac{S_{1}(k)}{S_{2}(k)}\approx 0$*and *$\frac{S_{1}(k)-k_{i}k_{j}}{\min(k_{i},k_{j})S_{1}(k)}\approx 0$

### Protein-protein interaction and gene co-expression network applications

#### Drosophila and yeast protein-protein network

To illustrate our results, we computed network concepts in module networks based on Drosophila and yeast protein-protein interaction (PPI) networks downloaded from BioGrid [31]. As described before, we defined the modules as branches of the hierarchical clustering dendrogram, see Figure 1.

Of the 1371 proteins in the Drosophila PPI network, 862 were clustered into 28 modules, and the remaining proteins grouped into an improper (grey) module. The module sizes of the proper modules range from 10 to 96, mean 30.79, median 23, and interquartile range 24.

Of the 2292 proteins in the yeast PPI network, 2050 were clustered into 44 proper modules, and the remaining proteins grouped into an improper module. The module sizes of the proper modules range from 10 to 219, mean 46.59, median 24, and interquartile range 38.8.

#### Yeast gene co-expression networks

We now illustrate our theoretical results using gene co-expression networks that have been used by many authors, e.g. [11,21,32]. Gene co-expression networks are constructed on the basis of microarray data from the transcriptional response of cells to changing conditions. There is evidence that genes with similar expression profiles are more likely to encode interacting proteins [33,34].

In gene co-expression networks, nodes correspond to gene expression profiles. The corresponding adjacency matrix is determined from a measure of co-expression between the genes. In the examples below, we will use the absolute value of the Pearson correlation coefficient between the gene expression profiles to measure co-expression between gene pairs. As detailed at the end of the Methods section, one can transform the Pearson correlation matrix into an adjacency matrix by hard thresholding or soft thresholding. Hard thresholding results in an unweighted network and soft thresholding results in a weighted network [21]. We applied our methods to a yeast cell cycle microarray data comprised of 44 microarrays and 2001 genes. This dataset recorded gene expression levels during different stages of cell cycles in yeasts and has been widely used before to illustrate clustering methods [35].

Of the 2001 genes (microarray probesets) in the weighted yeast gene co-expression network, 1081 were clustered into 8 proper modules. The module sizes of the proper modules range from 53 to 308, mean 135.1, median 101.5, and interquartile range 69.3. To facilitate a comparison between the weighted and the unweighted gene co-expression networks, we used the module assignment of the weighted network for the unweighted network as well. It turns out that the module assignment is highly preserved between the weighted and the unweighted gene co-expression networks, see Figures 1C) and 1D).

#### Functional annotation of modules

Since the scope of this paper is a mathematical and topological analysis of module networks, we defined modules without regard to external gene ontology information. Also we do not provide an in-depth analysis of the biological meaning of the network modules. But we briefly mention that there is indirect evidence that most of the resulting modules are biologically meaningful. We used the functional gene annotation tools from the Database for Annotation, Visualization and Integrated Discovery (DAVID) [36] to test for both enriched biochemical pathways and subcellular compartmentalization. We find that most modules are significantly enriched with known gene ontologies. A functional enrichment analysis for each network application is provided. For the Drosophila PPI network, [see Additional file 3]; for the yeast PPI network, [see Additional file 4]; for the weighted and unweighted yeast gene co-expression networks, [see Additional file 5].

#### Empirical relationships in 4 different networks

In accordance with Observation 2, we find a close relationship (*R*^2 ^≥ 0.6) between the fundamental network concepts and their approximate CF-based analogs. Specifically, we relate the network density, centralization, heterogeneity and clustering coefficients to their approximate CF-based analogs in Figures 2 (Drosophila PPI network), Figure 3 (yeast PPI network), and Figure 4 (weighted and unweighted yeast gene co-expression networks).

In accordance with Observation 3, we find a close relationship (*R*^2 ^≥ 0.6) between the mean clustering coefficient *mean *(***ClusterCoef ***) and (1 + *Heterogeneity*^2^)^2 ^× *Density*. Further, we find a close relationship between *TopOverlap*_[1]*j *_and (*Centralization *+ *Density*)(1 + *Heterogeneity*^2^), see Figures 5 (Drosophila PPI network), Figure 6 (yeast PPI network), and Figure 7 (weighted and unweighted yeast gene co-expression networks).

We find that our theoretical observations fit better in the weighted- than in the unweighted yeast gene co-expression network.

### Network concepts and module size

Since the number of genes inside a module (module size) varies greatly among the modules, it is natural to wonder whether the reported relationships between network concepts are due to the underlying module sizes. We find that the relationship between fundamental network concepts and their approximate CF-based analogs remains highly significant even after correcting for module sizes [see Additional file 2]. The same holds for the relationships between network concepts. Thus, none of the reported relationships is trivially due to module sizes. But we find that many network concepts depend on the underlying module size. We find that large modules are less factorizable than small modules: there is a strong negative correlation between module factorizability *F*(*A*) and module size. We also find that fundamental network concepts (e.g. density) depend on module size in our applications. For the factorizability, density, centralization, heterogeneity and mean clustering coefficient, the correlation coefficients with module size are -0.84, -0.46, -0.17, 0.26, and -0.36 in Drosophila PPI module networks; they are -0.55, -0.52, 0.05, 0.5, and -0.44 in yeast PPI module networks; they are -0.93, -0.52, -0.82, 0.27, and -0.55 in weighted yeast gene co-expression module networks; they are -0.86, -0.77, -0.56, 0.87, and -0.85 in unweighted yeast gene co-expression module networks. A more detailed analysis is presented in the Additional files [see Additional file 2].

### A simple exactly factorizable network example: constant network

A simple, exactly factorizable network is given by an adjacency matrix *A *with constant adjacencies (*a*_*ij *_= *b*, *b *∈ (0, 1]). The adjacency matrix is exactly factorizable since *a*_*ij *_= *CF*_*i*_*CF*_*j *_where *CF*_*i *_= $\sqrt{b}$. This network can be interpreted as the expected adjacency matrix of an Erdös-Rényi network [37]. One can easily derive the following expressions for the fundamental network concepts: *Connectivity*_*i *_= (*n *- 1)*b*, *Density *= *b*, *Centralization *= 0, *Heterogeneity *= 0, *ClusterCoef*_*i *_= *b *and *TopOverlap*_*ij *_= *b*.

Since *A *is exactly factorizable, the fundamental network concepts equal their CF-based analogs. However, the *approximate *CF-based concepts are different from their exact counterparts, see Table 3. For reasonably large values of *n*, the fundamental network concepts are very close to their approximate CF-based analogs. This illustrates Observation 2. With the results in Table 3, one can easily verify Observation 3 and equation (16) in this example.

**Table 3:** Network concepts in the constant Erdös-Rényi network.

Network Concepts	Fundamental	Approximate CF-based
*Connectivity*_*i*_	(*n *- 1)*b*	*nb*
*Density*	*b*	$b\frac{n}{n-1}$
*Centralization*	0	0
*Heterogeneity*	0	0
*TopOverlap*_*ij*_	*b*	$b\frac{nb+1}{(n-1)b+1}$
*ClusterCoef*_*i*_	*b*	*b*

### Example: block diagonal adjacency matrix

In the following, we will consider a block diagonal adjacency matrix where each block has constant adjacencies, i.e.

(17) $$A=\left(\begin{array}{cccccccc} 1 & b_{1} & \cdots & b_{1} & 0 & 0 & \cdots & 0\\ b_{1} & 1 & \cdots & b_{1} & 0 & 0 & \cdots & 0\\ \vdots & \vdots & \ddots & \vdots & \vdots & \vdots & \ddots & \vdots \\ b_{1} & b_{1} & \cdots & 1 & 0 & 0 & \cdots & 0\\ 0 & 0 & \cdots & 0 & 1 & b_{2} & \cdots & b_{2}\\ 0 & 0 & \cdots & 0 & b_{2} & 1 & \cdots & b_{2}\\ \vdots & \vdots & \ddots & \vdots & \vdots & \vdots & \ddots & \vdots \\ 0 & 0 & \cdots & 0 & b_{2} & b_{2} & \cdots & 1 \end{array}\right).$$

We assume that the first and second blocks have dimensions *n*_1 _× *n*_1 _and *n*_2 _× *n*_2_, respectively. Such a block diagonal matrix can be interpreted as a network with two distinct modules. Setting *n*_2 _= 0 results in the simple constant adjacency matrix, which we considered before.

We denote by ***f***_1 _= (1, 1,..., 1, 0, 0, ..., 0) the vector whose first *n*_1 _components equal 1 and the remaining components equal 0. Similarly, we define ***f***_2 _= (0, 0, ...,0, 1, 1, ..., 1) = ***1 ***- ***f***_1_. To simplify the calculation of the conformity, we further assume that

(18) $$\frac{n_{2}(n_{2}-1)b_{2}^{2}}{n_{1}(n_{1}-1)b_{1}^{2}}<1.$$

Then the conformity is uniquely defined by

$$CF=\sqrt{b_{1}}f_{1},$$

as one can show using equations (36) and (37) in the appendix. Further, using Proposition 10 in the appendix, one can show that the factorizability is given by

(19) $$F(A)=\frac{n_{1}(n_{1}-1)b_{1}^{2}}{n_{1}(n_{1}-1)b_{1}^{2}+n_{2}(n_{2}-1)b_{2}^{2}}.$$

In particular, if *n*_1 _≈ *n*_2 _and *b*_1 _= *b*_2_, i.e. if the adjacency matrix is comprised of two nearly identical blocks, the factorizability is *F*(*A*) ≈ 1/2. Similarly, one can show that if the matrix *A *is comprised of *B *identical blocks, then *F*(*A*) ≈ 1/*B*.

This block diagonal network allows one to arrive at explicit formulas for fundamental-, CF-based-, and approximate CF-based network concepts, see Table 4.

**Table 4:** Network concepts in the simulated block-diagonal network.

Concept	Fundamental	CF-based	Approx CF-based
*Connectivity*_*i*_	$(n_{1}-1)b_{1}Ind_{i\leq n_{1}}+(n_{2}-1)b_{2}Ind_{i>n_{1}}$	$(n_{1}-1)b_{1}Ind_{i\leq n_{1}}$	$n_{1}b_{1}Ind_{i\leq n_{1}}$
*Density*	$\frac{n_{1}(n_{1}-1)b_{1}+n_{2}(n_{2}-1)b_{2}}{\left(n_{1}+n_{2})(n_{1}+n_{2}-1\right)}$		$\frac{n_{1}^{2}b_{1}}{\left(n_{1}+n_{2})(n_{1}+n_{2}-1\right)}$
*Centralization*	$\frac{n_{2}((n_{1}-1)b_{1}+(n_{2}-1)b_{2})}{\left(n_{1}+n_{2}-1)(n_{1}+n_{2}-2\right)}$	$\frac{(n_{1}-1)n_{2}b_{1}}{\left(n_{1}+n_{2}-1)(n_{1}+n_{2}-2\right)}$	$\frac{n_{1}n_{2}b_{1}}{\left(n_{1}+n_{2}-1)(n_{1}+n_{2}-2\right)}$
*Heterogeneity*	$\sqrt{\frac{\left(n_{1}+n_{2})[n_{1}{\left(n_{1}-1\right)}^{2}b_{1}^{2}+n_{2}{\left(n_{2}-1\right)}^{2}b_{2}^{2}\right]}{{\left[n_{1}(n_{1}-1)b_{1}+n_{2}(n_{2}-1)b_{2}\right]}^{2}}-1}$	$\sqrt{\frac{n_{2}}{n_{1}}}$	$\sqrt{\frac{n_{2}}{n_{1}}}$
*TopOverlap*_*ij*_	$b_{1}Ind_{i,j\leq n_{1}}+b_{2}Ind_{i,j>n_{1}}$	$b_{1}Ind_{i,j\leq n_{1}}$	$b_{1}\frac{n_{1}b_{1}+1}{(n_{1}-1)b_{1}+1}Ind_{i,j\leq n_{1}}$
*ClusterCoef*_*i*_	$b_{1}Ind_{i\leq n_{1}}+b_{2}Ind_{i>n_{1}}$	$b_{1}Ind_{i\leq n_{1}}$	$b_{1}Ind_{i\leq n_{1}}$

The indicator function *Ind*(·) takes on the value 1 if the condition is satisfied and 0 otherwise.

In the following, we study the relationship between fundamental network concepts and their approximate CF-based analogs in the limit when the block diagonal network becomes approximately factorizable. Specifically, we calculate network concepts in the limit *b*_2 _→ 0 when *n*_1_, *n*_2 _and *b*_1 _are kept fixed. Under this assumption, *b*_2_→ 0 is equivalent to *F*(*A*) → 1. Then, one can easily show that

$$\begin{array}{lll} \underset{F(A)\to 1}{\lim}Connectivity_{i} & = & \frac{n_{1}-1}{n_{1}}Connectivity_{CF,app,i},\\ \underset{F(A)\to 1}{\lim}Density & = & \frac{n_{1}-1}{n_{1}}Density_{CF,app},\\ \underset{F(A)\to 1}{\lim}Centralization & = & \frac{n_{1}-1}{n_{1}}Centralization_{CF,app},\\ \underset{F(A)\to 1}{\lim}Heterogeneity & = & Heterogeneity_{CF,app},\\ \underset{F(A)\to 1}{\lim}TopOverlap_{ij} & = & \frac{(n_{1}-1)b_{1}+1}{n_{1}b_{1}+1}TopOverlap_{CF,app,ij},\\ \underset{F(A)\to 1}{\lim}ClusterCoef_{i} & = & ClusterCoef_{CF,app,i}. \end{array}$$

For reasonably large values of *n*_1 _(say *n*_1 _> 20), these limits illustrate Observation 2. Similarly, one can easily verify Observation 3 and equation (16) in the case when the factorizability *F*(*A*) is close to 1 and *n*_1 _is reasonably large.

## Discussion

This paper does not describe a new software or method for constructing networks. Instead, it presents theoretical results which clarify the mathematical relationship between network concepts in module networks. A deeper understanding of network concepts may guide the data analyst on how to construct and use networks in practice. Our results will pertain to any network that is approximately factorizable irrespective of its construction method. While the term 'factorizable' network is new, numerous examples of these types of networks can be found in the literature, e.g. [38]. A recent physical model for experimentally determined protein-protein interactions is exactly factorizable [39]. In that model, the 'affinity' *a*_*ij *_between proteins *i *and *j *is the product of the corresponding conformities. The conformities are approximately given by *CF*_*i *_= *exp*(-*K*_*i*_) where *K*_*i *_is the number of hydrophobic residues in the *i*-th protein. Another related example is an exactly factorizable random network model for which the edges between pairs of nodes are drawn according to a linking probability function [40,41].

We find that in many applications, the conformity is highly related to the first eigenvector of the adjacency matrix. The idea of using a variant of the singular value decomposition for decomposing an adjacency matrix has been proposed by several authors [42-45]. However, we prefer to define the conformity as a maximizer of the factorizability function $F_{A}(v)=1-\frac{\sum_{i}\sum_{j\neq i}{\left(a_{ij}-v_{i}v_{j}\right)}^{2}}{\sum_{i}\sum_{j\neq i}{\left(a_{ij}\right)}^{2}}$ for the following reasons: First, the factorizability satisfies that *F*_*A*_(***CF***) = 1 if, and only if, *A *is exactly factorizable network with *a*_*ij *_= *CF*_*i*_*CF*_*j*_. Second, we prefer to define the conformity without reference to the diagonal elements *a*_*ii *_of the adjacency matrix. Third, the definition naturally fits within the framework of least squares factor analysis where conformity can be interpreted as the first factor [46]. An algorithm for computing the conformity in general networks is presented in the appendix. While network analysis focuses on the adjacency matrix, factor analysis takes as input a correlation or covariance matrix. In module networks, the first factor (conformity) corresponds to a normalized connectivity measure, see equation (16). Future research could explore the network interpretation of higher order factors.

The topological structure of complex networks has been the focus of numerous studies, e.g. [7,8,16-18,20,38,47]. Here we explore the structure of special types of networks, which we refer to as module networks.

To derive results for factorizable module networks, we define several novel terms including a measure of network factorizability *F*(*A*), conformity, CF-based network concepts, approximate CF-based network concepts.

The first result (Observation 1) uses both PPI and gene co-expression network data to show empirically that subnetworks comprised of module nodes are often approximately factorizable. This insight could be interesting to researchers who develop module detection methods. Approximate factorizability is a very stringent structural assumption that is not satisfied in general networks. While modules in gene co-expression networks tend to be approximately factorizable if the corresponding expression profiles are highly correlated, the situation is more complicated for modules in PPI networks: only after replacing the original adjacency matrix by a 'smoothed out' version (the topological overlap matrix), do we find that the resulting modules are approximately factorizable.

The second result (Observation 2) shows that fundamental network concepts are approximately equal to their approximate CF-based analogs in approximately factorizable networks (e.g. modules). While fundamental network concepts are defined with respect to the adjacency matrix, approximate CF-based network concepts are defined with respect to the conformity vector. The close relationship between fundamental and approximate CF-based concepts in module networks can be used to provide an intuitive interpretation of network concepts in modules. We demonstrate that these high correlations between module concepts remain significant even after adjusting the analysis for differences in module size [see Additional file 2].

The third result (Observation 3) shows that the mean clustering coefficient is determined by the density and the network heterogeneity in approximately factorizable networks. Further, the topological overlap between two nodes is determined by the maximum of their respective connectivities and the heterogeneity. Thus, seemingly disparate network concepts satisfy simple and intuitive relationships in these special but biologically important types of networks.

The fourth result (Observation 4) is that in approximately factorizable networks, fundamental network concepts can be expressed as simple functions of the connectivity. Under mild assumptions, we argue that the clustering coefficient and the topological overlap matrix can be approximated by simple functions of the connectivity.

Our empirical data also highlight how network concepts differ between subnetworks of 'proper' modules and the subnetwork comprised of improper (grey) module nodes, see Table 1. For all applications, we find that proper modules have high factorizability, high density, high mean conformity. Based on our theoretical derivations, it comes as no surprise that proper modules also have a high average clustering coefficient and a high centralization when compared to the improper module. But we find no difference in heterogeneity between proper and improper module networks.

As a consequence of approximate factorizability, network concepts with disparate meanings in social network theory are closely related in module networks. Our results shed some light on the relationship between network concepts traditionally used by social scientists (e.g. centralization, heterogeneity) and concepts used by systems biologists (e.g. topological overlap). For example, equation (13) shows that in module networks, the topological overlap between a hub gene and other module genes is related to the centralization.

## Conclusion

Using several protein-protein interaction and gene co-expression networks, we provide empirical evidence that subnetworks comprised of module nodes often satisfy an important structural property, which we call 'approximate factorizability'. In these types of networks, simple relationships exist between seemingly disparate network concepts. Several network concepts with very different meanings in general networks turn out to be highly correlated across modules. These results are pertinent for systems biology since a biological pathways may correspond to an approximately factorizable module network.

## Methods

### The adjacency matrix and notation

We study the properties of an adjacency matrix (network) *A *that satisfies the following three conditions:

(A.1) *A *is symmetric and has dimension *n × n*.

(A.2) The entries of *A *are bounded within [0, 1], that is, 0 ≤ *a*_*ij *_≤ 1 for all 1 ≤ *i*,*j *≤ *n*.

(A.3) The diagonal elements of *A *are all 1, that is, *a*_*ii *_= 1 for all 1 ≤ *i *≤ *n*.

#### Matrix and vector notation

We will make use of the following notations. We denote by ***e***_*i *_the unit vector whose *i*-th entry equals 1 and by ***1 ***the 'one' vector whose components all equal 1. The Frobenius matrix norm is denoted by ${\left\|M\right\|}_{F}=\sqrt{\sum_{i}\sum_{j}m_{ij}^{2}}$. The transpose of a matrix or vector is denoted by the superscript ^τ ^. For any real number *p*, we use the notation *M*^*p *^and ***v***^*p *^to denote the element-wise power of a matrix *M *and a vector ***v ***respectively. We define the function *S*_*p*_(·) for a vector ***v ***as *S*_*p*_(***v***) = ∑_*i *_$v_{i}^{p}$ = (***v***^*p*^)^*τ *^***1***. Further denote by *I *the identity matrix and by diag(***v***^2^) a diagonal matrix with its *i*-th diagonal component given by $v_{i}^{2}$,*i *= 1, ...,*n*. We define the maximum function max(*M*) as the maximum entry of matrix *M *and max(***v***) as the maximum entry of the vector ***v***. Similarly we define the minimum function min(·). Also, we define *mean*(***v***) = *S*_1_(***v***)/*n *and *variance*(***v***) = *S*_2_(***v***)/*n *- (*S*_1_(***v***)/*n*)^2^.

### Uniqueness of the conformity for an exactly factorizable network

One can easily show that the vector ***CF ***is not unique if an exactly factorizable network contains only *n *= 2 nodes. However, for *n > *2 the conformity is uniquely defined when dealing with a weighted network where *a*_*ij *_*> *0.

Specifically, we prove the following statement. If *A *is an *n *× *n *(*n *≥ 3) dimensional adjacency matrix with positive entries (*a*_*ij *_> 0), then the system of equations in (7) has at most one solution ***CF ***with positive entries. If the solution exists, it is given by

(20) $$CF_{i}={\left(\frac{p_{i}}{{\left(\prod_{m=1}^{n}p_{m}\right)}^{1/(2(n-1))}}\right)}^{\frac{1}{n-2}},$$

where $p_{i}=\prod_{j=1}^{n}a_{ij}$ denotes the 'product connectivity' of the *i*-th node.

Proof: by assumption, we have *a*_*ij *_= *CF*_*i*_*CF*_*j *_for a positive vector ***CF ***and *n *≥ 3. Multiplying both sides of equation (7) yields $\prod_{m}\prod_{l\neq m}a_{lm}=\prod_{m}\prod_{l\neq m}CF_{l}CF_{m}={\left(\prod_{l=1}^{n}CF_{l}\right)}^{2(n-1)}$. Since $\prod_{l=1}^{n}CF_{l}$ is positive, we find $\prod_{l=1}^{n}CF_{l}={\left(\prod_{m}\prod_{l\neq m}a_{lm}\right)}^{\frac{1}{2(n-1)}}$. Similarly, eliminating the *i*-th row and column from *A *yields $\prod_{l\neq i}CF_{l}={\left(\prod_{m\neq i}\prod_{l\neq m,i}a_{lm}\right)}^{\frac{1}{2(n-1)}}={\left(\prod_{m}\prod_{l\neq m}a_{lm}/{\left(\prod_{l\neq i}a_{li}\right)}^{2}\right)}^{\frac{1}{2(n-1)}}$. Since $CF_{i}=\prod_{l=1}^{n}CF_{l}/\prod_{l\neq i}CF_{l}$, we conclude that *CF*_*i *_is uniquely defined by

$$\begin{array}{c} CF_{i}=\frac{{\left(\prod_{m}\prod_{l\neq m}a_{lm}\right)}^{\frac{1}{2(n-1)}}}{{\left(\prod_{m\neq i}\prod_{l\neq m,i}a_{lm}\right)}^{\frac{1}{2(n-1)}}}=\frac{{\left(\prod_{m}\prod_{l\neq m}a_{lm}\right)}^{\frac{1}{2(n-1)}}}{{\left(\prod_{m}\prod_{l\neq m}a_{lm}\right)}^{\frac{1}{2(n-2)}}}{\left(\prod_{m=1}^{n}a_{im}\right)}^{\frac{1}{n-2}}\\ ={\left(\frac{p_{i}}{{\left(\prod_{m=1}^{n}p_{m}\right)}^{1/(2(n-1))}}\right)}^{\frac{1}{n-2}}. \end{array}$$

### Network concept functions and fundamental network concepts

In general, we define a *network concept function *to be a tensor valued function (e.g. the connectivity vector) that takes a square matrix (e.g. the network adjacency matrix) as input.

Denote by *M *= [*m*_*ij*_] a general *n *× *n *matrix. Then we will study the following network concept functions:

(21) $$\begin{array}{ccc} Connectivity_{i}(M) & = & \sum_{j}m_{ij}=e_{i}^{\tau }M1,\\ Density(M) & = & \frac{\sum_{i}\sum_{j}m_{ij}}{n(n-1)},\\ Centralization(M) & = & \frac{n}{n-2}\left(\frac{\max(M1)}{n-1}-Density(M)\right),\\ Heterogeneity(M) & = & \sqrt{\frac{n(1^{\tau }MM1)}{{\left(1^{\tau }M1\right)}^{2}}-1},\\ TopOverlap_{ij}(M) & = & \frac{e_{i}^{\tau }MMe_{j}+e_{i}^{\tau }Me_{j}}{\min\{e_{i}^{\tau }M1,e_{j}^{\tau }M1\}+1-e_{i}^{\tau }Me_{j}},\\ ClusterCoef_{i}(M) & = & \frac{e_{i}^{\tau }MMMe_{i}}{e_{i}^{\tau }MB_{M}Me_{i}}, \end{array}$$

where the components of matrix *B*_*M *_in the denominator of the clustering coefficient function are given by *b*_*ij *_= 1 if *i *≠ *j *and *b*_*ii *_= *Ind*(*m*_*ii *_> 0). Here the indicator function *Ind*(·) takes on the value 1 if the condition is satisfied and 0 otherwise.

For the sake of brevity, we study only a limited selection of network concept functions and do not claim that these are more important than others studied in the literature. Our general formalism for relating fundamental network concepts to their approximate CF-based analogs should allow the reader to adapt our derivations to alternative concepts as well.

Now we are ready to define the fundamental network concepts that are studied in this article.

**Definition 5 (Fundamental Network Concept) ***The fundamental network concepts of a network A are defined by evaluating the network functions (equation (21)) on A *- *I, i.e.*

*FundamentalNetworkConcept *= *NetworkConcept*(*A *- *I*).

As special cases of this definition, we find the following concepts. The **connectivity **(also known as degree) of the *i*-th node is given by

$$k_{i}=Connectivity_{i}(A-I)=\sum_{j\neq i}a_{ij}.$$

The **line density **[13] equals the mean adjacency, i.e

(22) $$Density(A-I)=\frac{\sum_{i}\sum_{j\neq i}a_{ij}}{n(n-1)}=\frac{S_{1}(k)}{n(n-1)}=\frac{mean(k)}{n-1}.$$

For notational convenience, we sometimes omit the reference to the adjacency matrix and simply write *Density *to denote the fundamental network concepts.

The normalized connectivity **centralization **(also known as degree centralization) [14] is given by

(23) $$Centralization(A-I)=\frac{n}{n-2}\left(\frac{\max(k)}{n-1}-Density\right)=\frac{n}{\left(n-2)(n-1\right)}(\max(k)-mean(k)).$$

Our definition of the network **heterogeneity **equals the coefficient of variation of the connectivity distribution, i.e.

(24) $$Heterogeneity(A-I)=\frac{\sqrt{variance(k)}}{mean(k)}=\sqrt{\frac{nS_{2}(k)}{S_{1}{\left(k\right)}^{2}}-1}.$$

Note that *Heterogeneity*(b * *M*) = *Heterogeneity*(*M*) for a scalar *b *≠ 0.

The **clustering coefficient **of node *i *is a density measure of local connections, or 'cliquishness' [19,20]. Specifically,

(25) $$ClusterCoef_{i}=ClusterCoef_{i}(A-I)=\frac{n_{i}}{\pi_{i}}=\frac{\sum_{l\neq i}\sum_{m\neq i,l}a_{il}a_{lm}a_{mi}}{\left\{{\left(\sum_{l\neq i}a_{il}\right)}^{2}-\sum_{l\neq i}a_{il}^{2}\right\}}.$$

The **topological overlap **between nodes *i *and *j *reflects their relative interconnectedness. It is defined by

(26) $$TopOverlap_{ij}=TopOverlap_{ij}(A-I)=\frac{l_{ij}+a_{ij}}{\min\{k_{i},k_{j}\}+1-a_{ij}},$$

where *l*_*ij *_= ∑_*u*≠*i*,*j*_*a*_*iu*_*a*_*uj*_.

### Network concepts in exactly factorizable networks

In the following, we will present explicit formulas for the fundamental network concepts in Definition 5 when the adjacency matrix *A *is exactly factorizable, i.e. if *a*_*ij *_= *CF*_*i*_*CF*_*j*_. We define the CF-based adjacency matrix as follows

(27) *A*_*CF *_:= ***CF CF***^*τ *^- *diag*(***CF***^2^) + *I*,

where *diag*(***CF***^2^) denotes the diagonal matrix with diagonal elements $CF_{i}^{2}$, *i *= 1 ...*n*. Then one can easily show that for exactly factorizable networks

(28) $$\begin{array}{ccc} A & = & A_{CF,}\\ NetworkConcept(A-I) & = & NetworkConcept(A_{CF}-I). \end{array}$$

Using our definition of network concept functions in equations (21), one can easily derive the following formulas for *NetworkConcept*(*A*_*CF *_- *I*) in terms of the quantities *S*_*p*_(***CF***) = ∑_*i*_$CF_{i}^{p}$.

(29) $$\begin{array}{lll} Connectivity_{i}(A_{CF}-I) & = & CF_{i}S_{1}(CF)-CF_{i}^{2},\\ Density(A_{CF}-I) & = & \frac{S_{1}{\left(CF\right)}^{2}-S_{2}(CF)}{n(n-1)},\\ Centralization(A_{CF}-I) & = & \frac{n}{n-2}\left(\frac{\max(Connectivity(A_{CF}-I))}{n-1}-Density(A_{CF}-I)\right),\\ Heterogeneity(A_{CF}-I) & = & \sqrt{\frac{n(S_{2}(CF)S_{1}{\left(CF\right)}^{2}-2S_{3}(CF)S_{1}(CF)+S_{4}(CF))}{{\left(S_{1}{\left(CF\right)}^{2}-S_{2}(CF)\right)}^{2}}-1},\\ ClusterCoef_{i}(A_{CF}-I) & = & \frac{\left(S_{2}(CF)-{CF_{i}^{2})}^{2}-(S_{4}(CF)-CF_{i}^{4}\right)}{{\left(S_{1}(CF)-CF_{i}\right)}^{2}-(S_{2}(CF)-{CF_{i}^{2})}^{2}}.\\ TopOverlap_{ij}(A_{CF}-I) & = & \frac{CF_{i}CF_{j}(S_{2}(CF)-CF_{i}^{2}-CF_{j}^{2})+CF_{i}CF_{j}}{\min(CF_{i}(S_{1}(CF)-CF_{i}),CF_{j}(S_{1}(CF)-CF_{j}))+1-CF_{i}CF_{j}} \end{array}$$

### Approximate CF-based network concepts in general networks

When *A*_*CF *_- *I *is used as input of a network concept function, it gives rise to a CF-based network concept as detailed in the following

**Definition 6 (CF-based Network Concepts) ***Assume that the conformity vector ****CF ****can be defined for a general adjacency matrix A. Then the CF-based network concepts are defined by evaluating the network concept functions on A*_*CF *_- *I *= ***CF CF***^*τ *^- *diag*(***CF***^2^)*, i.e.*

*NetworkConcept*_*CF *_:= *NetworkConcept*(*A*_*CF *_- *I*).

By definition, fundamental network concepts are equal to their CF-based analogs if *A *is exactly factorizable.

In the following, we define *approximate *CF-based analogs of the fundamental network concepts. The theoretical advantage of these approximate CF-based concepts is that they satisfy simple relationships. Define the *approximate CF-based adjacency matrix *as follows

(30) *A*_*CF*,*app *_= ***CF CF***^*τ*^.

Note that only the diagonal elements differ between *A*_*CF*,*app *_and *A*_*CF *_. We define the approximate CF-based network concepts by using *AC*_*F*,*app *_as input of the network concept functions as detailed in the following

**Definition 7 (Approximate CF-based Network Concepts) ***The approximate CF-based network concepts of a network A with conformity ****CF ****are defined by evaluating the network functions (equations (21)) on A*_*CF*,*app *_= ***CF CF***^*τ*^*, i.e.*

*NetworkConcept*_*CF*,*app *_:= *NetworkConcept*(*A*_*CF*,*app*_).

### In approximately factorizable networks, fundamental network concepts are approximately equal to their approximate CF-based analogs

Here we will provide a heuristic derivation of Observation 2. Since the components of ***CF ***are positive, one can easily show that *S*_4_(***CF***) ≤ *S*_2_(***CF***)^2^. For many large, exactly factorizable networks, the ratio *S*_4_(***CF***)*/S*_2_(***CF***)^2 ^is close to 0. Since *S*_4_(***CF***)/*S*_2_(***CF***)^2 ^= ${\left\|(A_{CF}-I)-A_{CF,app}\right\|}_{F}^{2}/{\left\|A_{CF,app}\right\|}_{F}^{2}$, this implies that *A*_*CF *_- *I *≈ *A*_*CF*,*app*_. Since the network concept functions are continuous functions, this implies *NetworkConcept*(*A*_*CF *_- *I*) ≈ *NetworkConcept*(*A*_*CF*,*app*_). These derivations are summarized in the following

**Observation 8 (Approximate Formulas for CF-based Concepts) ***If S*_4_(***CF***)/*S*_2_(***CF***)^2 ^≈ 0, *then*

(31) *NetworkConcept*(*A*_*CF *_- *I*) ≈ *NetworkConcept*(*A*_*CF*,*app*_).

In particular, for exactly factorizable networks (i.e. *A *- *I *= *A*_*CF *_- *I*), this means that the fundamental network concepts can be approximated by their approximate CF-based analogs.

In our real data applications, we show empirically that equation (31) holds even in networks that satisfy the assumptions of Observation 8 only approximately.

In the appendix (equation (43)), we define a measure of network factorizability as follows

(32) $$F(A)=1-\frac{{\left\|\left(A-I)-(A_{CF}-I\right)\right\|}_{F}^{2}}{{\left\|A-I\right\|}_{F}^{2}},$$

Thus, in approximately factorizable networks (i.e. *F*(*A*) close to 1), *A *- *I *can be approximated by *A*_*CF *_- *I*. For a continuous network functions, this implies

*NetworkConcept*(*A *-*I*) ≈ *NetworkConcept*(*A_CF_*- *I*),

i.e. the fundamental network concepts are approximately equal to their CF-based analogs in approximately factorizable networks. Observation 8 states that

*NetworkConcept*(*A*_*CF *_-*I*) ≈ *NetworkConcept*(*A*_*CF*,*app*_).

Combining the last two equations leads to *NetworkConcept*(*A *- *I*) ≈ *NetworkConcept*(*A*_*CF*,*app*_). These derivations are summarized as follows.

In approximately factorizable networks, the fundamental network concepts are approximately equal to their approximate CF-based analogs, i.e.

*FundamentalNetworkConcept *≈ *NetworkConcept*_*CF*,*app*_.

### Construction of gene co-expression networks

Gene co-expression networks are constructed from microarray data that measures the transcriptional response of cells to changing conditions. We consider the case of *n *genes with gene expression profiles across *m *microarray samples. Thus, the gene expression profiles are given by an *n *× *m *matrix

(33) ***X ***= [*x*_*ij*_] = (***x***_1 _***x***_2 _ ... ***x***_*n*_)^*τ*^, *i *= 1, ..., *n*; *j *= 1, ..., *m*,

where the *i*-th row $x_{i}^{\tau }$ is the transcriptional responses of the *i*-th gene.

Recently, several groups have suggested thresholding the pairwise Pearson correlation coefficient *cor*(***x***_*i*_, ***x***_*j*_) in order to arrive at gene co-expression networks, which are sometimes referred to as 'relevance' networks [11,32]. In these networks, a node corresponds to the gene expression profile of a given gene. The corresponding adjacency matrix is determined from a measure of co-expression between the genes. In the examples below, we will use the absolute value of the Pearson correlation coefficient between the gene expression profiles to measure co-expression.

To transform the co-expression measure into an adjacency, one can make use of an *adjacency function*. The choice of the adjacency function determines whether the resulting network will be weighted (soft-thresholding) or unweighted (hard-thresholding). The adjacency function is a monotonically increasing function that maps the interval [0, 1] into [0, 1]. A widely used adjacency function is the signum function which implements 'hard' thresholding involving the threshold parameter *τ*. Specifically,

(34) *a*_*ij *_= *Signum*(|*cor*(***x***_*i*_, ***x***_*j*_)|, *τ*) = *Ind*(|*cor*(***x***_*i*_, ***x***_*j*_)| ≥ *τ*),

where the indicator function *Ind*(·) takes on the value 1 if the condition is satisfied and 0 otherwise. Hard thresholding using the signum function leads to intuitive network concepts (e.g., the node connectivity equals the number of direct neighbors), but it may lead to a loss of information: if τ has been set to 0.8, there will be no connection between two nodes if their similarity equals 0.79.

To avoid the disadvantages of hard thresholding, we proposed a 'soft' thresholding approach that raises the absolute value of the correlation to the power *β *≥ 1 [21], i.e.

(35) *a*_*ij *_= *Power*(|*cor*(***x***_*i*_, ***x***_*j*_)|, *β*) = |*cor*(***x***_*i*_, ***x***_*j*_)|^*β*^.

In our yeast cell cycle gene co-expression network analysis, we followed the analysis steps described in [21]. Briefly, we used the 2001 most varying and connected genes. Next, we used the power adjacency function with *β *= 7 (equation (35)) to construct a weighted gene co-expression network and the signum adjacency function with *τ *= 0.65 (equation (34)) to construct an unweighted network.

Using our R software tutorial, the reader can easily verify that our conclusions are highly robust with respect to a) different ways of constructing co-expression networks and b) different ways of constructing modules.

## Availability and requirements

An R implementation and the data can be obtained from the internet: http://www.genetics.ucla.edu/labs/horvath/ModuleConformity/ModuleNetworks

## Appendix: node conformity and factorizability of a general network

Equation (20) provides an explicit formula for the conformity of a weighted, exactly factorizable network. For a general, non-factorizable network, we describe here how to compute the conformity by optimizing an objective function. In the following, we assume a general *n *× *n *adjacency matrix *A *where *n *> 2. Let ***v ***= (*v*_1_,*v*_2_, ...,*v*_*n*_)^*τ *^be a vector of length *n*. We could define the conformity as a vector v* that *minimizes *the following objective function *f*(***v***) = ∑_*i *_∑_*j*≠*i*_(*a*_*ij *_- *v*_*i*_*v*_*j*_)^2^. But instead, we find the following equivalent formulation as a maximization problem more useful since it naturally gives rise to a measure of factorizability.

Specifically, we define the objective function

(36) $$F_{A}(v):=1-\frac{\sum_{i}\sum_{j\neq i}{\left(a_{ij}-v_{i}v_{j}\right)}^{2}}{\sum_{i}\sum_{j\neq i}{\left(a_{ij}\right)}^{2}}=1-\frac{{\left\|A-I+diag(v^{2})-vv^{\tau }\right\|}_{F}^{2}}{{\left\|A-I\right\|}_{F}^{2}}.$$

It is clear that *F*_*A*_(***CF***) = 1 for an exactly factorizable network with *a*_*ij *_= *CF*_*i*_*CF*_*j *_for *i *≠ *j*. Note that *F*_*A*_(***v***) ≤ 1 and *F*_*A*_(***0 ***) = 0. One can easily show that if v* maximizes *F*_*A*_(***v***), then -v* also maximizes *F*_*A*_(***v***). Further, all components of v* must have the same sign since otherwise, flipping the sign of the negative components leads to a higher value of *F*_*A*_(***v***). This leads us to the following

**Definition 9 (Conformity, Factorizability) ***We define the conformity ****CF ****as the vector with non-negative entries that maximizes F*_*A*_(***v***)*. If there is more than one such maximizer, then a maximizer closest to ****k/***$\sqrt{S_{1}(k)}$*is chosen. Further, we define the factorizability F*(*A*) *as the corresponding maximum value F*_*A*_(***CF***)*.*

Our definition of the conformity is a generalization of Definition 7 since *F*(*A*) = 1 if, and only if, *A *is exactly factorizable with *a*_*ij *_*= CF*_*i*_*CF*_*j *_*for i *≠ *j*. The advantages of this Definition are briefly described in the discussion section.

In general, *F*_*A*_(***v***) may have multiple maximizers as can be demonstrated with the block diagonal simulated example (equation (17)) by choosing *n*_1 _= *n*_2 _and *b*_1 _= *b*_2_. By forming the first derivative of the factorizability function *F*_*A*_(***v***) in terms of *v*_*i*_, one can show that a local maximum satisfies

(37) $$\sum_{j\neq i}a_{ij}CF_{j}=CF_{i}\sum_{j\neq i}CF_{j}^{2},$$

i.e.

(38) $$(A-I+diag(CF^{2}))CF=CF||CF|{|}_{2}^{2}.$$

**Proposition 10 (Expressions for the Factorizability) ***If the conformity vector ****CF ****of the adjacency matrix A exists, then the factorizability F*(*A*) *is given by*

(39) $$F(A)=\frac{{\left\|A_{CF}-I\right\|}_{F}^{2}}{{\left\|A-I\right\|}_{F}^{2}}=\frac{S_{2}{\left(CF\right)}^{2}-S_{4}(CF)}{{\left\|A-I\right\|}_{F}^{2}}.$$

**Proof **Since $F(A)=1-\frac{{\left\|(A-I)+diag{\left(CF\right)}^{2}-CF\;CF^{\tau }\right\|}_{F}^{2}}{{\left\|A-I\right\|}_{F}^{2}}$, it will be sufficient to show that ${\left\|\left(A-I)-(A_{CF}-I\right)\right\|}_{F}^{2}={\left\|A-I\right\|}_{F}^{2}-{\left\|A_{CF}-I\right\|}_{F}^{2}$. From the definition of the Frobenius norm of a matrix *B*, one can show that ${\left\|B\right\|}_{F}^{2}=trace(B^{\tau }B)$ where the trace of a matrix is the sum of its diagonal elements. Thus, ${\left\|\left(A-I)-(A_{CF}-I\right)\right\|}_{F}^{2}={\left\|A-I\right\|}_{F}^{2}+{\left\|A_{CF}-I\right\|}_{F}^{2}-2\times trace((A-I)(A_{CF}-I))$. Using equation (38), we find that *trace*((*A *- *I*)(*A*_*CF *_- *I*)) = *tr*((*A *- *I*)***CF CF***^*τ*^) - *tr*((*A *- *I*)*diag*(***CF***^2^)) = ***CF***^*τ*^(*A *- *I*)***CF ***= ${\left\|A_{CF}-I\right\|}_{F}^{2}$. Thus, ${\left\|\left(A-I)-(A_{CF}-I\right)\right\|}_{F}^{2}={\left\|A-I\right\|}_{F}^{2}-{\left\|A_{CF}-I\right\|}_{F}^{2}$ The remainder of the proof is straightforward.

Equation (38) suggests that the conformity is an eigenvector of the 'hat' adjacency matrix

$$\hat{A}$$ := *A *- *I *+ *diag*(***CF***^2^).

An algorithm for computing the conformity is based on the following

**Lemma 11 ***If A denotes a symmetric real matrix with eigenvalues d*_1_, ..., *d*_*n *_*sorted according to their absolute values, i.e*., |*d*_1_| ≥ |*d*_2_| ≥ ... ≥ |*d*_*n*_|*, and the corresponding orthonormal eigenvectors are denoted by ****u***_1_, ..., ***u***_*n*_*, then *${\left\|A-vv^{\tau }\right\|}_{F}^{2}$*is minimized at *v* = $\sqrt{\left|d_{1}\right|}$***u***_1_.

The proof can be found in Horn and Johnson [49].

Denote by ***CF***(*i *- 1) an estimate of the conformity ***CF***. Next define

(40) $$\hat{A}$$(*i *- 1) = *A *- *I *+ *diag*(***CF***(*i *- 1)^2^).

Define a new estimate of the conformity by

(41) $$CF(i)=\sqrt{{\hat{d}}_{1}(i-1)}{\hat{u}}_{1}(i-1),$$

where ${\hat{d}}_{1}$ (*i *- 1) and ${\hat{u}}_{1}$ (*i *- 1) denote the largest eigenvalue and corresponding unit length eigenvector of $\hat{A}$ (*i *- 1). One can easily show that all the components of ${\hat{u}}_{1}$ (*i *- 1) must have the same sign and we assume without loss of generality non-negative components. Lemma 11 with *A *= $\hat{A}$ (*i *- 1) implies that

$$\begin{array}{c} {\left\|A-I+diag(CF{\left(i-1\right)}^{2})-CF(i-1)CF{\left(i-1\right)}^{\tau }\right\|}_{F}^{2}\\ \geq {\left\|A-I+diag(CF{\left(i-1\right)}^{2})-CF(i)CF{\left(i\right)}^{\tau }\right\|}_{F}^{2}. \end{array}$$

Considering the diagonal elements, one can easily show that

$$\begin{array}{c} {\left\|A-I+diag(CF{\left(i-1\right)}^{2})-CF(i)CF{\left(i\right)}^{\tau }\right\|}_{F}^{2}\\ \geq {\left\|A-I+diag(CF{\left(i\right)}^{2})-CF(i)CF{\left(i\right)}^{\tau }\right\|}_{F}^{2}. \end{array}$$

Thus, we arrive at the following

(42) *F*_*A*_(***CF***(*i*)) ≥ *F*_*A*_(***CF***(*i *- 1)),

which suggests a monotonic algorithm for computing ***CF***. Equation (16) suggests to choose ***k/***$\sqrt{S_{1}(k)}$ as a starting value of the algorithm. These comments give rise to the following

**Definition 12 (Algorithmic Definition of Conformity, Factorizability) ***For a general network A, set ****CF***(1) = ***k/***$\sqrt{S_{1}(k)}$*and apply the monotonic iterative algorithm described by equations (40) and (41). If the limit ****CF***(∞) *exists, we define it as the conformity ****CF ***= ***CF***(∞)*. Further, we define the network factorizability as*

(43) $$F(A)=1-\frac{{\left\|\left(A-I)-(A_{CF}-I\right)\right\|}_{F}^{2}}{{\left\|A-I\right\|}_{F}^{2}}.$$

Note that the conformity satisfies equation (38) by definition of convergence. One can easily show that 0 ≤ *F*(*A*) ≤ 1. Further, one can easily show that *F*(*A*) = 1 if, and only if, *A *is exactly factorizable with *a*_*ij *_= *CF*_*i*_*CF*_*j*_, i.e. *A *- *I *= *A*_*CF *_- *I*.

The algorithm described by equations (40) and (41) is monotonic (equation (42)). It is a special case of an algorithm described in [46] for fitting a least squares factor analysis model with one factor. Theoretical properties of the algorithm are discussed in [46] and [48].

We find that for most real networks, the conformity is highly related to the first eigenvector of the adjacency matrix, i.e. the conformity vector ***CF ***is roughly equal to $\sqrt{d_{1}}$***u***_1 _where *d*_1 _is the largest eigenvalue of *A *and ***u***_1 _is the corresponding unit length eigenvector with positive components.

## Supplementary Material

Additional file 1 Complete list of network concepts in the modules. An extended version of Table 1. Click here for file

Additional file 2 Network concepts and module size. Descriptions of how module concepts are related to module sizes in the Drosophila PPI, yeast PPI networks, and yeast gene co-expression networks. Click here for file

Additional file 3 Functional enrichment analysis (gene ontology) of the Drosophila PPI modules (DAVID software). Click here for file

Additional file 4 Functional enrichment analysis (gene ontology) of the yeast PPI modules (DAVID software). Click here for file

Additional file 5 Functional enrichment analysis (gene ontology) of the yeast gene co-expression modules (DAVID software). Click here for file
