# Supplementary material for: Understanding network concepts in modules
Source: BMC Syst Biol. 2007 Jun 4;1:24. doi: 10.1186/1752-0509-1-24 (PMC3238286; doi:10.1186/1752-0509-1-24)
Supplement: Additional file 2 — Network concepts and module size. Descriptions of how module concepts are related to module sizes in the Drosophila PPI, yeast PPI networks, and yeast gene co-expression networks. [file 1752-0509-1-24-S2.pdf]

Methods Supplement: The Dependence of Network Concept on Module Size

Jun Dong and Steve Horvath*

*Correspondence: shorvath@mednet.ucla.edu

Here we describe empirical studies regarding the relationship between network concepts and module size. This additional file accompanies our main article 'Understanding network concepts in modules'.

We report results for the 4 networks described in our main article: Drosophila PPI, yeast PPI, yeast weighted gene co-expression network, and the corresponding unweighted co-expression network. A description of the datasets and the modules can be found in the main article.

We use scatter plots and correlation coefficients to relate network concepts to the underlying module sizes, see Figures 1, 2, 3 and 4. The colors of the points correspond to the module colors. Grey denotes the improper module comprised of genes outside any proper module.

Each section corresponds to one dataset, and each subsection corresponds to one approximation equation by either its approximate CF-based analogue or by one of our two observations (Observation 2 and 3 in the main article). The first 4 subsections study the relationships between the fundamental network concepts and their approximate CF-based analogues for density, centralization, heterogeneity and clustering coefficients. The next 2 subsections study the relationships of

$$mean(\mathbf{ClusterCoef}) \approx \left(1 + Heterogeneity^2\right)^2 \times Density,$$

and

$$TopOverlap_{[1]j} \approx \frac{k_{[1]}}{n}(1 + Heterogeneity^2),$$

which are equations (11) and (14) in the main article.

We show that the reported relationships between network concepts remain significant even after adjusting the analysis for module size. To study adjust for module size, we use multiple linear regression models. For example, we regress the fundamental network concept on its approximate CF-based analogue and module size. While module size is sometimes a significant predictor, we find that it is less significant than the approximate CF-based analog. The fact that the approximate CF-based analog remains a highly significant covariate in the multiple regression model demonstrates that the relationship between fundamental network concepts and CF-based approximations is not trivially due to the underlying module size.

We also use multiple linear regression models to argue that the relationship among fundamental network concepts remains highly significant even after adjusting for module size.

In the last subsection, we report the relationship between module size and factorizability $F(A)$. We find that larger modules tend to be less factorizable than smaller modules.

The data and a corresponding software tutorial can be downloaded from the following webpage: http://www.genetics.ucla.edu/labs/horvath/ModuleConformity/ModuleNetworks

1 Drosophila PPI Network

The relationships between fundamental network concepts and module sizes for Drosophila protein-protein networks are illustrated in Figure 1.

1.1 Drosophila PPI: Density and the Approximate CF-based Density

```
lm(formula = Density ~ Approx. CF-based Density + Module Size)

Coefficients:
                        Estimate Std. Error t value Pr(>|t|)
(Intercept)             6.190e-03  5.060e-03   1.223    0.232
Approx. CF-based Density 9.181e-01  2.025e-02  45.339   <2e-16
Module Size             3.719e-06  1.940e-05   0.192    0.850
---
Residual standard error: 0.008304 on 26 degrees of freedom
Multiple R-Squared: 0.9902,     Adjusted R-squared: 0.9894
F-statistic:  1312 on 2 and 26 DF,  p-value: < 2.2e-16
```

Note that the most significant predictor of density is approximate CF-based density and module size is not significant in this model. Also note that the regression model explains 99 percent of the variance (R-squared measure). The relationship between fundamental network concepts and the covariates is highly significant ($p < 2.2e\text{-}16$).

1.2 Drosophila PPI: Centralization and the Approximate CF-based Centralization

```
lm(formula = Centralization ~ Approx. CF-based Centralization + Module Size)

Coefficients:
                                Estimate Std. Error t value Pr(>|t|)
(Intercept)                    4.211e-04  1.386e-02   0.030    0.976
Approx. CF-based Centralization 8.664e-01  5.704e-02  15.189 1.92e-14
Module Size                    5.173e-05  6.399e-05   0.808    0.426
---
Residual standard error: 0.03012 on 26 degrees of freedom
Multiple R-Squared: 0.9019,     Adjusted R-squared: 0.8943
F-statistic: 119.5 on 2 and 26 DF,  p-value: 7.842e-14
```

Note that the most significant predictor of centralization is approximate CF-based centralization and module size is not significant in this model.

1.3 Drosophila PPI: Heterogeneity and the Approximate CF-based Heterogeneity

```
lm(formula = Heterogeneity ~ Approx. CF-based Heterogeneity + Module Size)

Coefficients:
                               Estimate Std. Error t value Pr(>|t|)
(Intercept)                   3.845e-02  2.567e-02   1.498    0.146
Approx. CF-based Heterogeneity 7.977e-01  6.326e-02  12.611 1.38e-12
Module Size                   7.995e-05  1.018e-04   0.785    0.439
---
Residual standard error: 0.04808 on 26 degrees of freedom
Multiple R-Squared: 0.8691,     Adjusted R-squared: 0.859
F-statistic: 86.3 on 2 and 26 DF,  p-value: 3.319e-12
```

Note that the most significant predictor of heterogeneity is the approximate CF-based heterogeneity; module size is not significant in this model.

1.4 Drosophila PPI: Clustering Coefficient and the Approximate CF-based Clustering Coefficient

```
lm(formula = Clustering Coefficients ~ Approx. CF-based Clustering Coefficients + Module Size)

Coefficients:
                                          Estimate Std. Error t value Pr(>|t|)
(Intercept)                              5.103e-03  6.267e-03   0.814  0.41565
Approx. CF-based Clustering Coefficients  9.883e-01  1.936e-02  51.050  < 2e-16
Module Size                              3.824e-05  1.231e-05   3.105  0.00194
---
Residual standard error: 0.05114 on 1368 degrees of freedom
Multiple R-Squared: 0.8725,     Adjusted R-squared: 0.8723
F-statistic:  4681 on 2 and 1368 DF,  p-value: < 2.2e-16
```

Note that the most significant predictor of the clustering coefficients is the approximate CF-based clustering coefficients. While module size is significant, note that the corresponding Student T statistic (3.105) is much smaller than that of the approx CF-based clustering coefficient (51.05). This suggest that the significant p-value (0.00194) of the module size is partly due to the large number of observations (1371).

1.5 Drosophila PPI: Clustering Coefficient and the Approximation by Equation (11)

In our article, we derive the following relationship in approximately factorizable module networks.

$$mean(\mathbf{ClusterCoef}) \approx \left(1 + Heterogeneity^2\right)^2 \times Density, \tag{1}$$

Here we study whether this relationship remains significant after adjusting the analysis for module size. In the following regression model, the covariate 'Approx. Clustering Coefficients' denotes $\left(1 + Heterogeneity^2\right)^2 \times Density$. We find

```
lm(formula = Clustering Coefficients ~ Approx. Clustering Coefficients + Module Size)

Coefficients:
                                 Estimate Std. Error t value Pr(>|t|)
(Intercept)                    -1.394e-03  6.454e-03  -0.216    0.829
Approx. Clustering Coefficients  1.055e+00  2.089e-02  50.517  < 2e-16
Module Size                     4.952e-05  1.263e-05   3.922 9.23e-05
---
Residual standard error: 0.0515 on 1368 degrees of freedom
Multiple R-Squared: 0.8707,     Adjusted R-squared: 0.8706
F-statistic:  4608 on 2 and 1368 DF,  p-value: < 2.2e-16
```

Note that the most significant predictor of the clustering coefficient is the approximate CF-based clustering coefficient.

1.6 Drosophila PPI: TOM with the Hub Node and the Approximation by Equation (14)

In our article, we argue that the topological overlap between the most highly connected node and all other nodes is approximately constant. Specifically, if we denote the index of the most highly connected node by [1] and its connectivity by $k_{[1]} = max(\boldsymbol{k})$, then

$$TopOverlap_{[1]j} \approx \frac{k_{[1]}}{n}(1 + Heterogeneity^2). \tag{2}$$

Here we study whether this relationship remains significant after adjusting the analysis for module size.

```
lm(formula = TOM with the Hub ~ Approx. TOM with the Hub + Module Size)

Coefficients:
                                  Estimate Std. Error t value Pr(>|t|)
(Intercept)                      2.925e-02  7.308e-03   4.003  6.6e-05
Approx. TOM with the Hub         8.329e-01  1.347e-02  61.824  < 2e-16
Module Size                     -4.356e-05  1.347e-05  -3.234  0.00125
---
Residual standard error: 0.06198 on 1368 degrees of freedom
Multiple R-Squared: 0.9084,     Adjusted R-squared: 0.9083
F-statistic:  6785 on 2 and 1368 DF,  p-value: < 2.2e-16
```

Note the difference in Student T test statistics.

1.7 Drosophila PPI: Factorizability

```
lm(formula = Factorizability ~ Module Size)

Coefficients:
                 Estimate Std. Error t value Pr(>|t|)
(Intercept)     0.8632108  0.0171460  50.345  < 2e-16
Module Size    -0.0013566  0.0001687  -8.041 1.22e-08
---
Residual standard error: 0.08173 on 27 degrees of freedom
Multiple R-Squared: 0.7054,     Adjusted R-squared: 0.6945
F-statistic: 64.65 on 1 and 27 DF,  p-value: 1.221e-08
```

This suggests that module size explains 70 percent of the variation in factorizability.

2 Yeast PPI Network

The relationships between fundamental network concepts and module sizes for yeast protein-protein networks are illustrated in Figure 2.

2.1 Density and the Approximate CF-based Density

```
lm(formula = Density ~ Approx. CF-based Density + Module Size)

Coefficients:
                          Estimate Std. Error t value Pr(>|t|)
(Intercept)              1.358e-02  4.680e-03   2.901  0.00589
Approx. CF-based Density 9.111e-01  1.220e-02  74.677  < 2e-16
Module Size              1.759e-05  3.032e-05   0.580  0.56495
---
Residual standard error: 0.00946 on 42 degrees of freedom
Multiple R-Squared: 0.9945,     Adjusted R-squared: 0.9943
F-statistic: 3817 on 2 and 42 DF,  p-value: < 2.2e-16
```

2.2 Yeast PPI: Centralization and the Approximate CF-based Centralization

```
lm(formula = Centralization ~ Approx. CF-based Centralization + Module Size)

Coefficients:
                                 Estimate Std. Error t value Pr(>|t|)
(Intercept)                    -1.301e-02  1.666e-02  -0.781    0.439
Approx. CF-based Centralization  8.612e-01  6.462e-02  13.327  < 2e-16
Module Size                     3.458e-04  7.559e-05   4.575 4.18e-05
---
Residual standard error: 0.02656 on 42 degrees of freedom
Multiple R-Squared: 0.8093,     Adjusted R-squared: 0.8002
F-statistic: 89.13 on 2 and 42 DF,  p-value: 7.708e-16
```

2.3 Yeast PPI: Heterogeneity and the Approximate CF-based Heterogeneity

```
lm(formula = Heterogeneity ~ Approx. CF-based Heterogeneity + Module Size)

Coefficients:
                               Estimate Std. Error t value Pr(>|t|)
(Intercept)                   0.0605356  0.0169827   3.565 0.000925
Approx. CF-based Heterogeneity 0.6590130  0.0393635  16.742  < 2e-16
Module Size                   0.0004087  0.0001355   3.016 0.004334
---
Residual standard error: 0.04574 on 42 degrees of freedom
Multiple R-Squared: 0.9023,     Adjusted R-squared: 0.8976
F-statistic: 193.9 on 2 and 42 DF,  p-value: < 2.2e-16
```

2.4 Yeast PPI: Clustering Coefficient and the Approximate CF-based Clustering Coefficient

```
lm(formula = Clustering Coefficients ~ Approx. CF-based Clustering Coefficients + Module Size)

Coefficients:
                                          Estimate Std. Error t value Pr(>|t|)
(Intercept)                              4.353e-02  6.243e-03   6.972 4.08e-12
Approx. CF-based Clustering Coefficients   8.329e-01  1.374e-02  60.620  < 2e-16
Module Size                              1.312e-05  2.372e-05   0.553     0.58
---
Residual standard error: 0.07391 on 2289 degrees of freedom
Multiple R-Squared: 0.701,      Adjusted R-squared: 0.7007
F-statistic:  2683 on 2 and 2289 DF,  p-value: < 2.2e-16
```

2.5 Yeast PPI: Clustering Coefficient and the Approximation by Equation (11)

```
lm(formula = Clustering Coefficients ~ Approx. Clustering Coefficients + Module Size)

Coefficients:
                                   Estimate Std. Error t value Pr(>|t|)
(Intercept)                       5.127e-02  5.462e-03   9.387   <2e-16
Approx. Clustering Coefficients    8.830e-01  1.288e-02  68.578   <2e-16
Module Size                      -3.384e-05  2.124e-05  -1.594    0.111
---
Residual standard error: 0.06826 on 2289 degrees of freedom
Multiple R-Squared: 0.7449,     Adjusted R-squared: 0.7447
F-statistic:  3342 on 2 and 2289 DF,  p-value: < 2.2e-16
```

2.6 Yeast PPI: Topological Overlap with the Hub Node and the Approximation by Equation (14)

```
lm(formula = TOM with the Hub ~ Approx. TOM with the Hub + Module Size)

Coefficients:
                                  Estimate Std. Error t value Pr(>|t|)
(Intercept)                      0.0147014  0.0077565   1.895   0.0582
Approx. TOM with the Hub         0.8847925  0.0117932  75.025  < 2e-16
Module Size                     -0.0001282  0.0000273  -4.697 2.80e-06
---
Residual standard error: 0.09416 on 2289 degrees of freedom
Multiple R-Squared: 0.7576,     Adjusted R-squared: 0.7574
F-statistic:  3577 on 2 and 2289 DF,  p-value: < 2.2e-16
```

2.7 Yeast PPI: Factorizability

```
lm(formula = Factorizability ~ Module Size)

Coefficients:
                  Estimate Std. Error t value Pr(>|t|)
(Intercept)      0.9041373  0.0239133  37.809  < 2e-16
Module Size     -0.0013721  0.0003201  -4.286 0.000101
---
Residual standard error: 0.1173 on 43 degrees of freedom
Multiple R-Squared: 0.2993,     Adjusted R-squared: 0.283
F-statistic: 18.37 on 1 and 43 DF,  p-value: 0.0001006
```

3 Yeast Weighted Gene Co-Expression Network

The relationships between fundamental network concepts and module sizes for the weighted yeast gene co-expression networks are illustrated in Figure 3.

3.1 Yeast WGCN: Density and the Approximate CF-based Density

```
lm(formula = Density ~ Approx. CF-based Density + Module Size)

Coefficients:
                          Estimate Std. Error t value Pr(>|t|)
(Intercept)              2.999e-03  8.303e-04   3.612   0.0112
Approx. CF-based Density  9.746e-01  7.506e-03 129.841 1.41e-11
Module Size             -1.754e-06  1.611e-06  -1.089   0.3181
---
Residual standard error: 0.00107 on 6 degrees of freedom
Multiple R-Squared: 0.9997,     Adjusted R-squared: 0.9997
F-statistic: 1.154e+04 on 2 and 6 DF,  p-value: 1.755e-11
```

3.2 Yeast WGCN: Centralization and the Approximate CF-based Centralization

```
lm(formula = Centralization ~ Approx. CF-based Centralization + Module Size)

Coefficients:
                                 Estimate Std. Error t value Pr(>|t|)
(Intercept)                    -9.070e-03  1.015e-02  -0.893    0.406
Approx. CF-based Centralization  9.488e-01  7.493e-02  12.662 1.49e-05
```

```
Module Size                     3.275e-06  1.070e-05   0.306    0.770
---
Residual standard error: 0.004601 on 6 degrees of freedom
Multiple R-Squared: 0.9881,     Adjusted R-squared: 0.9842
F-statistic: 249.5 on 2 and 6 DF,  p-value: 1.677e-06
```

3.3 Yeast WGCN: Heterogeneity and the Approximate CF-based Heterogeneity

```
lm(formula = Heterogeneity ~ Approx. CF-based Heterogeneity + Module Size)

Coefficients:
                                Estimate Std. Error t value Pr(>|t|)
(Intercept)                    0.2230348  0.1224581   1.821   0.1184
Approx. CF-based Heterogeneity  0.5768130  0.2122712   2.717   0.0348
Module Size                   -0.0002320  0.0001229  -1.888   0.1080
---
Residual standard error: 0.04598 on 6 degrees of freedom
Multiple R-Squared: 0.5838,     Adjusted R-squared: 0.4451
F-statistic: 4.208 on 2 and 6 DF,  p-value: 0.07209
```

3.4 Yeast WGCN: Clustering Coefficient and the Approximate CF-based Clustering Coefficient

```
lm(formula = Clustering Coefficients ~ Approx. CF-based Clustering Coefficients + Module Size)

Coefficients:
                                           Estimate Std. Error t value Pr(>|t|)
(Intercept)                               7.771e-03  2.435e-03   3.191 0.001441
Approx. CF-based Clustering Coefficients   8.953e-01  1.560e-02  57.379  < 2e-16
Module Size                               1.014e-05  2.631e-06   3.853 0.000120
---
Residual standard error: 0.02462 on 1998 degrees of freedom
Multiple R-Squared: 0.8244,     Adjusted R-squared: 0.8242
F-statistic:  4691 on 2 and 1998 DF,  p-value: < 2.2e-16
```

3.5 Yeast WGCN: Clustering Coefficient and the Approximation by Equation (11)

```
lm(formula = Clustering Coefficients ~ Approx. Clustering Coefficients + Module Size)

Coefficients:
                                  Estimate Std. Error t value Pr(>|t|)
(Intercept)                      1.412e-02  2.349e-03   6.010 2.20e-09
Approx. Clustering Coefficients   9.316e-01  1.635e-02  56.996  < 2e-16
Module Size                      1.003e-05  2.645e-06   3.791 0.000154
---
Residual standard error: 0.02472 on 1998 degrees of freedom
Multiple R-Squared: 0.823,      Adjusted R-squared: 0.8228
F-statistic:  4643 on 2 and 1998 DF,  p-value: < 2.2e-16
```

3.6 Yeast WGCN: TOM with the Hub Node and the Approximation by Equation (14)

```
lm(formula = TOM with the Hub ~ Approx. TOM with the Hub + Module Size)

Coefficients:
                                   Estimate Std. Error t value Pr(>|t|)
```

```
(Intercept)                        1.321e-02  6.039e-03   2.187   0.0288
Approx. TOM with the Hub           8.794e-01  2.343e-02  37.528   <2e-16
Module Size                       -1.024e-05  6.023e-06  -1.701   0.0892
---
Residual standard error: 0.04247 on 1998 degrees of freedom
Multiple R-Squared: 0.8111,     Adjusted R-squared: 0.8109
F-statistic:  4289 on 2 and 1998 DF,  p-value: < 2.2e-16
```

3.7 Yeast WGCN: Factorizability

```
lm(formula = Factorizability ~ Module Size)

Coefficients:
                   Estimate Std. Error t value Pr(>|t|)
(Intercept)       0.8151597  0.0345260  23.610 6.21e-08
Module Size      -0.0006750  0.0001014  -6.658 0.000288
---
Residual standard error: 0.07846 on 7 degrees of freedom
Multiple R-Squared: 0.8636,     Adjusted R-squared: 0.8441
F-statistic: 44.33 on 1 and 7 DF,  p-value: 0.0002883
```

4 Yeast Unweighted Gene Co-Expression Network

The relationships between fundamental network concepts and module sizes for the unweighted yeast gene co-expression networks are illustrated in Figure 4.

4.1 Yeast UGCN: Density and the Approximate CF-based Density

```
lm(formula = Density ~ Approx. CF-based Density + Module Size)

Coefficients:
                           Estimate Std. Error t value Pr(>|t|)
(Intercept)              -2.700e-03  3.613e-03  -0.747   0.4831
Approx. CF-based Density  9.899e-01  7.125e-03 138.922 9.38e-12
Module Size               1.351e-05  4.987e-06   2.708   0.0352
---
Residual standard error: 0.002426 on 6 degrees of freedom
Multiple R-Squared: 0.9999,     Adjusted R-squared: 0.9998
F-statistic: 2.369e+04 on 2 and 6 DF,  p-value: 2.030e-12
```

4.2 Yeast UGCN: Centralization and the Approximate CF-based Centralization

```
lm(formula = Centralization ~ Approx. CF-based Centralization + Module Size)

Coefficients:
                                  Estimate Std. Error t value Pr(>|t|)
(Intercept)                     -4.831e-02  4.182e-02  -1.155    0.292
Approx. CF-based Centralization  1.296e+00  1.076e-01  12.041 1.99e-05
Module Size                      5.179e-06  4.033e-05   0.128    0.902
---
Residual standard error: 0.02563 on 6 degrees of freedom
Multiple R-Squared: 0.9725,     Adjusted R-squared: 0.9634
F-statistic: 106.2 on 2 and 6 DF,  p-value: 2.073e-05
```

4.3 Yeast UGCN: Heterogeneity and the Approximate CF-based Heterogeneity

```
lm(formula = Heterogeneity ~ Approx. CF-based Heterogeneity + Module Size)

Coefficients:
                                Estimate Std. Error t value Pr(>|t|)
(Intercept)                    3.300e-01  1.446e-01   2.282   0.0626
Approx. CF-based Heterogeneity  3.480e-01  4.155e-01   0.838   0.4344
Module Size                   -1.045e-05  5.535e-04  -0.019   0.9856
---
Residual standard error: 0.07585 on 6 degrees of freedom
Multiple R-Squared: 0.7809,     Adjusted R-squared: 0.7079
F-statistic: 10.69 on 2 and 6 DF,  p-value: 0.01051
```

4.4 Yeast UGCN: Clustering Coefficient and the Approximate CF-based Clustering Coefficient

```
lm(formula = Clustering Coefficients ~ Approx. CF-based Clustering Coefficients + Module Size)

Coefficients:
                                   Estimate Std. Error t value Pr(>|t|)
(Intercept)                       2.956e-01  3.675e-02   8.044 1.47e-15
Approx. Clustering Coefficients    6.884e-01  5.238e-02  13.142  < 2e-16
Module Size                      -4.738e-05  3.036e-05  -1.560    0.119
---
Residual standard error: 0.1366 on 1998 degrees of freedom
Multiple R-Squared: 0.5971,     Adjusted R-squared: 0.5967
F-statistic:  1481 on 2 and 1998 DF,  p-value: < 2.2e-16
```

4.5 Yeast UGCN: Clustering Coefficient and the Approximation by Equation (11)

```
lm(formula = Clustering Coefficients ~ Approx. Clustering Coefficients + Module Size)

Coefficients:
                                  Estimate Std. Error t value Pr(>|t|)
(Intercept)                      2.733e-01  3.834e-02   7.127 1.43e-12
Approx. Clustering Coefficients   6.956e-01  5.281e-02  13.173  < 2e-16
Module Size                      5.688e-05  3.798e-05   1.498    0.134
---
Residual standard error: 0.1366 on 1998 degrees of freedom
Multiple R-Squared: 0.5973,     Adjusted R-squared: 0.5969
F-statistic:  1482 on 2 and 1998 DF,  p-value: < 2.2e-16
```

4.6 Yeast UGCN: TOM with the Hub Node and the Approximation by Equation (14)

```
lm(formula = TOM with the Hub ~ Approx. TOM with the Hub + Module Size)

Coefficients:
                                   Estimate Std. Error t value Pr(>|t|)
(Intercept)                       2.052e-01  7.309e-02   2.807  0.00505
Approx. TOM with the Hub          7.063e-01  6.152e-02  11.482  < 2e-16
Module Size                      -1.888e-04  6.041e-05  -3.125  0.00180
---
Residual standard error: 0.1949 on 1998 degrees of freedom
Multiple R-Squared: 0.7402,     Adjusted R-squared: 0.7399
F-statistic:  2846 on 2 and 1998 DF,  p-value: < 2.2e-16
```

4.7 Yeast UGCN: Factorizability

```
lm(formula = Factorizability ~ Module Size)

Coefficients:
                  Estimate Std. Error t value Pr(>|t|)
(Intercept)       0.7132678  0.0497065  14.350  1.9e-06
Module Size      -0.0006584  0.0001459  -4.511  0.00276
---
Residual standard error: 0.113 on 7 degrees of freedom
Multiple R-Squared: 0.7441,     Adjusted R-squared: 0.7075
F-statistic: 20.35 on 1 and 7 DF,  p-value: 0.002761
```

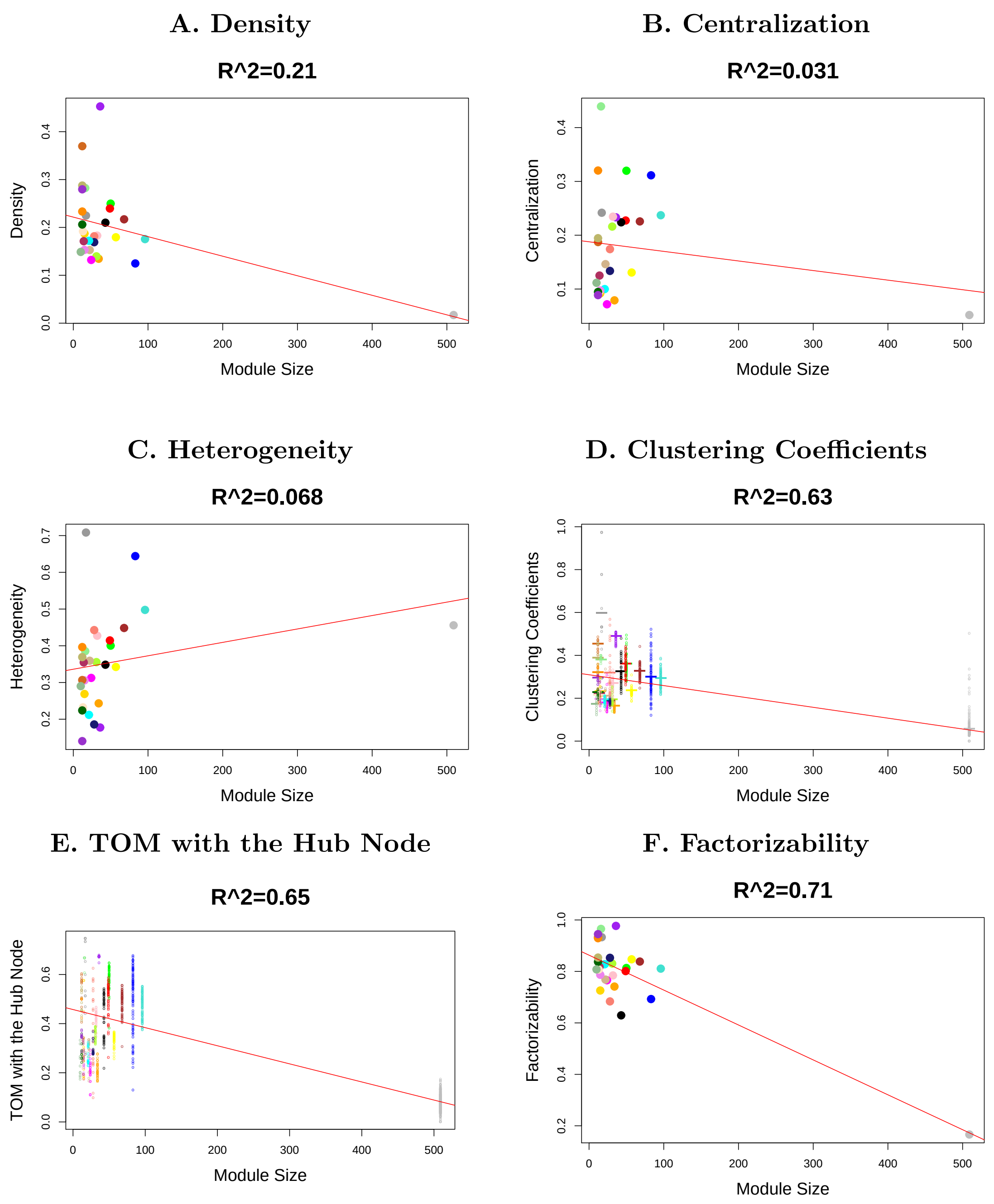

Figure 1: Drosophila PPI module networks: the relationship between fundamental network concepts $NetworkConcept(A - I)$ (y-axis) and the module sizes (x-axis).

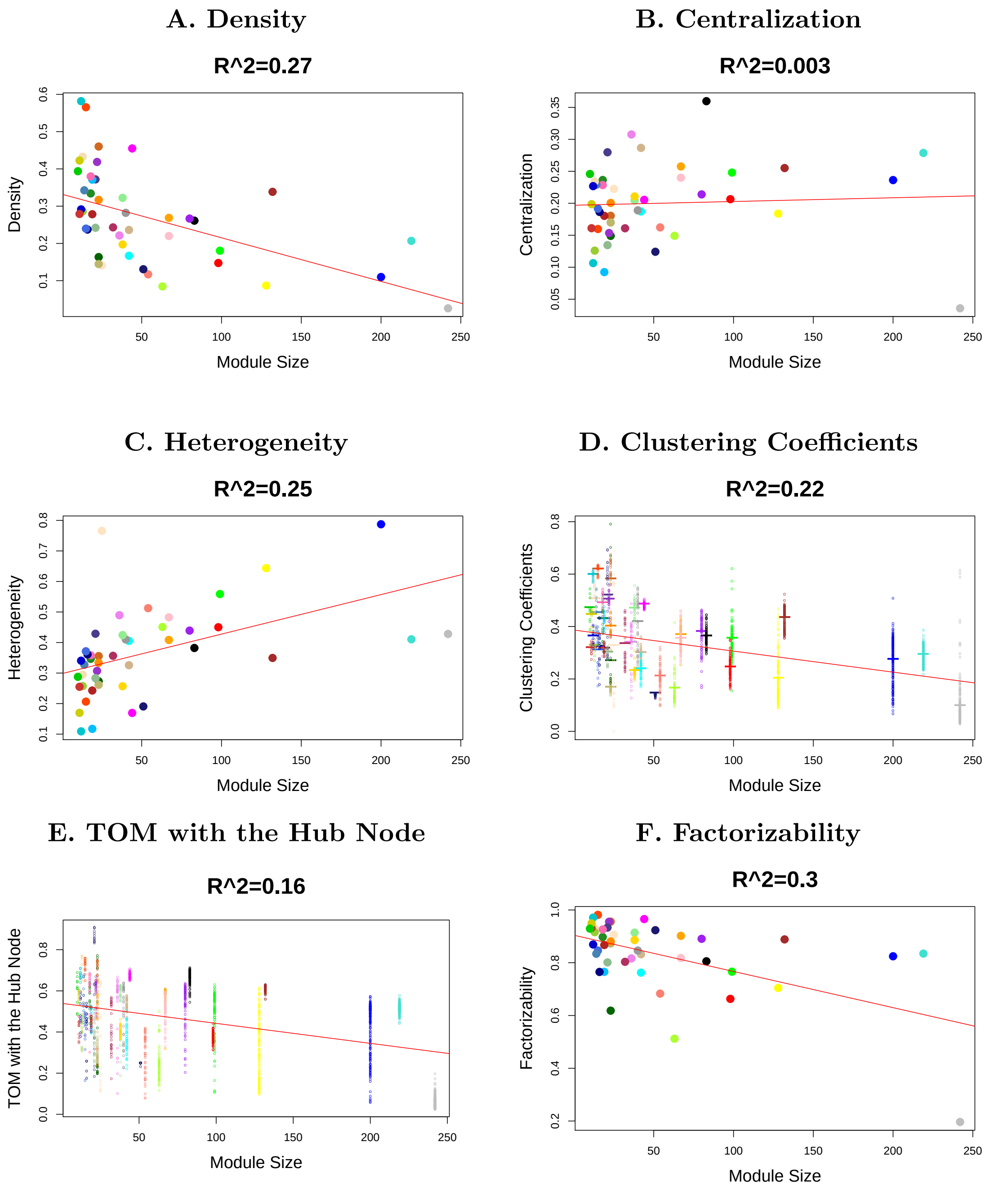

Figure 2: Yeast PPI module networks: the relationship between fundamental network concepts $NetworkConcept(A - I)$ (y-axis) and the module sizes (x-axis).

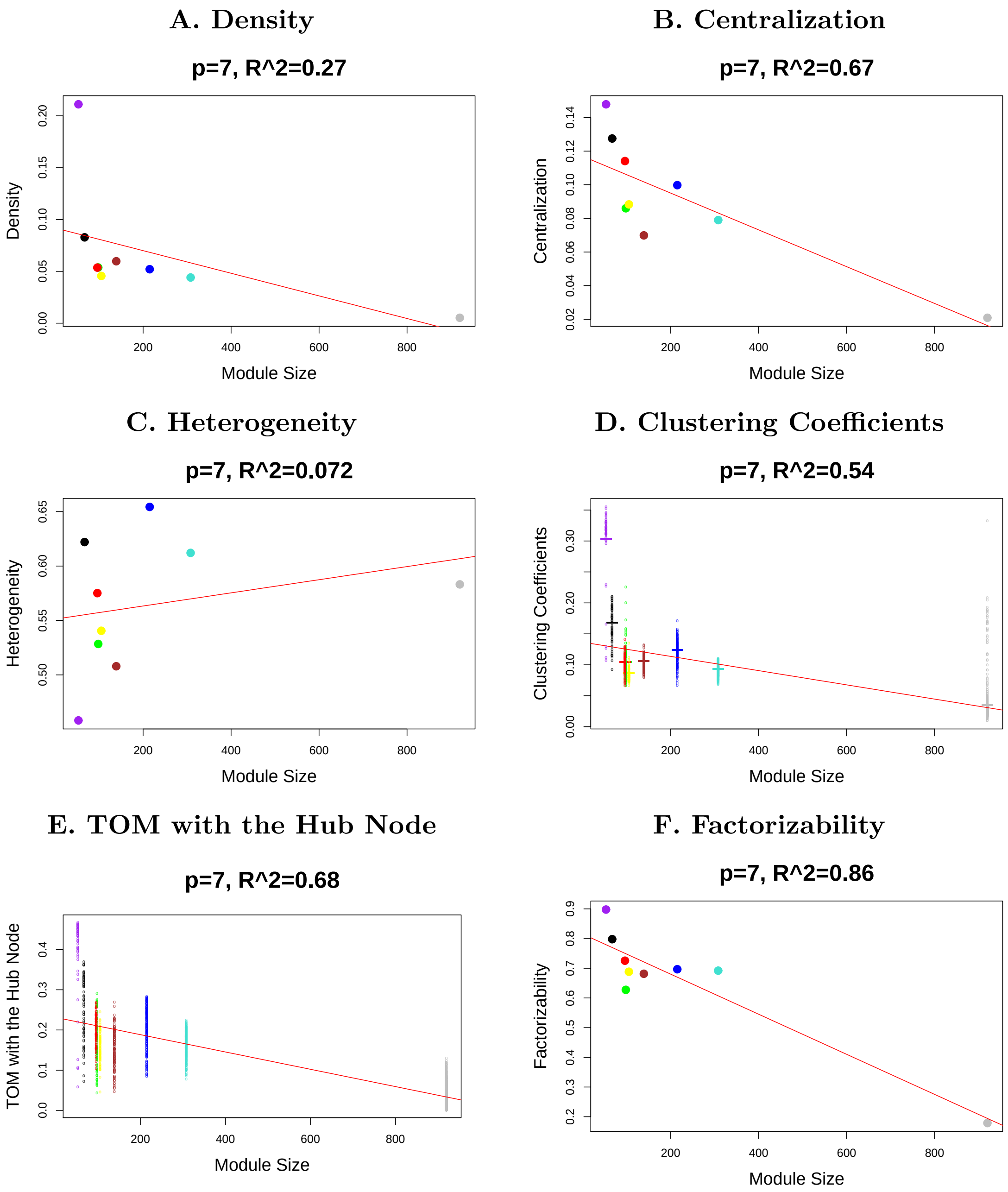

Figure 3: Yeast weighted gene co-expression module networks: the relationship between fundamental network concepts $NetworkConcept(A - I)$ (y-axis) and the module sizes (x-axis).

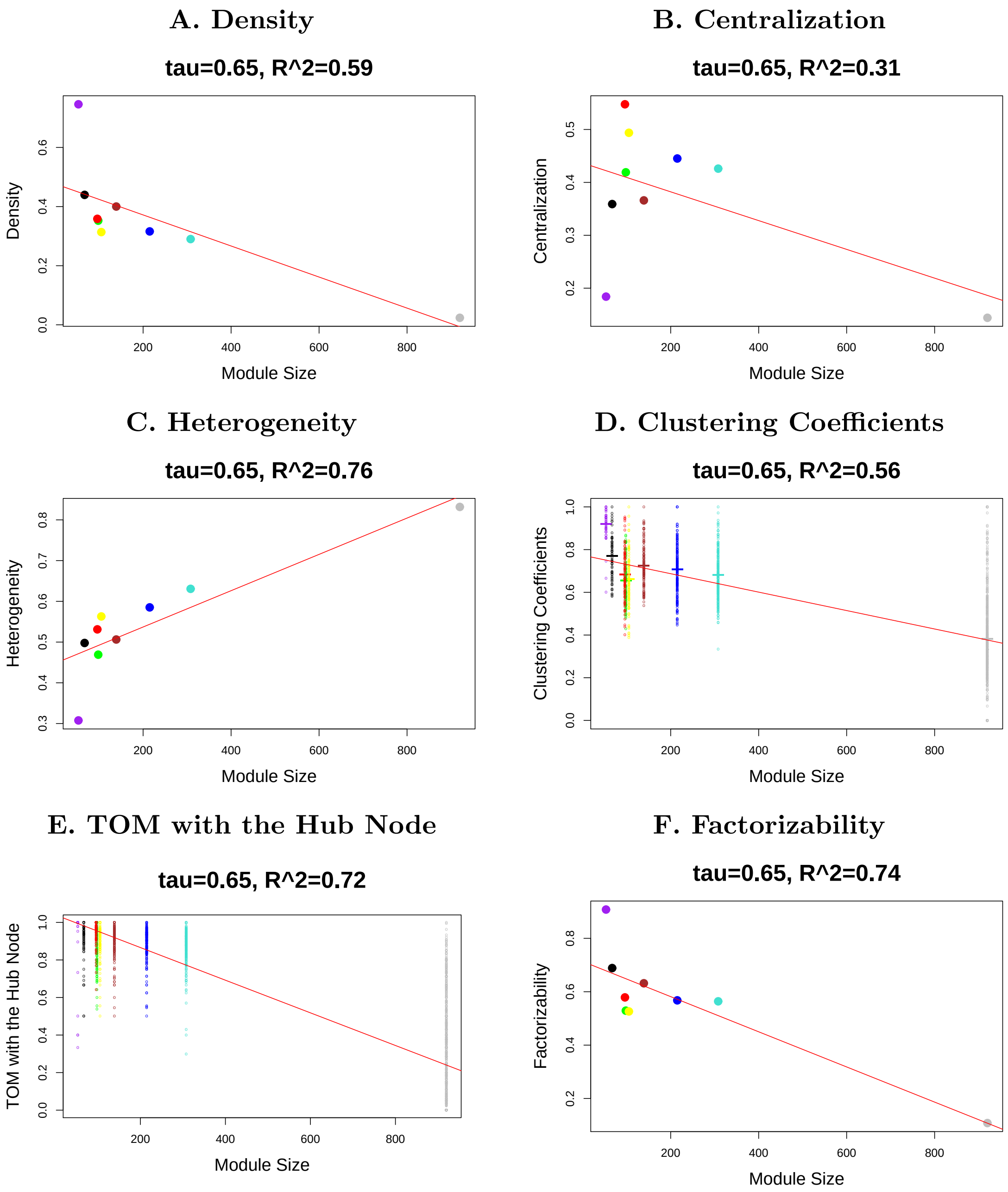

Figure 4: Yeast unweighted gene co-expression module networks: the relationship between fundamental network concepts $NetworkConcept(A - I)$ (y-axis) and the module sizes (x-axis).
